# Allogeneic Immune Cell Perfusion Inhibits the Growth of Vascularized 3D In Vitro Tumor Models, Induces Vascular Regression and Desmoplasia, but Promotes Tumor Cell Invasion

**DOI:** 10.1002/advs.202514361

**Published:** 2026-02-06

**Authors:** Alexandra Raab, Rasika Daware, Marcelo A. Szymanski de Toledo, Oskar Weber, Dimitris Kapsokalyvas, Twan Lammers, Horst Fischer, Federica De Lorenzi, Fabian Kiessling

**Affiliations:** ^1^ Institute For Experimental Molecular Imaging (ExMI) RWTH Aachen University Hospital Aachen Germany; ^2^ Department of Nanomedicine and Theranostics Institute for Experimental Molecular Imaging (ExMI) RWTH Aachen University Hospital Aachen Germany; ^3^ Department of Hematology Oncology, Hemostaseology, and Stem Cell Transplantation Faculty of Medicine RWTH Aachen University Aachen Germany; ^4^ Interdisciplinary Center for Clinical Research (IZKF) RWTH Aachen University Hospital Aachen Germany; ^5^ Department of Dental Materials and Biomaterials Research (ZWBF) RWTH Aachen University Hospital Aachen Germany; ^6^ Department of Medical Biosciences Radboud University Medical Center Nijmegen the Netherlands

**Keywords:** 3D vascularized tumors, bioreactors, immune‐stromal crosstalk

## Abstract

Advanced in vitro platforms incorporating vascularized tumors offer a promising approach to dissect biological interactions between cancer, stromal, and immune components, as well as for biological drug testing. Here, we employed a vascularized 3D bioreactor system to evaluate the impact of allogeneic peripheral blood mononuclear cell (PBMC) perfusion on breast cancer spheroids embedded within self‐organizing endothelial and stromal matrices. PBMC introduction results in rapid vascular regression, with reduced vessel density and interconnectivity of the self‐assembled networks. Tumor spheroids exposed to PBMC show increased apoptosis and pyroptosis, resulting in spheroid size reduction. Interestingly, this is accompanied by enhanced peripheral tumor cell proliferation and invasive dissemination into the surrounding matrix. While tumor spheroids alone stabilize vascular networks and activate stromal components, PBMC perfusion triggers further stromal activation and desmoplasia, indicating inflammation and immune‐mediated cytotoxicity. This approach demonstrates the multifaceted impact of allogeneic immune cell perfusion, including tumor suppression, vascular regression, stromal activation, and invasive tumor behavior, collectively reshaping the tumor microenvironment through innate immune‐driven inflammation. These findings emphasize the importance of accounting for donor mismatch and innate immune activation in designing translationally relevant vascularized tumor models, and they support the development of autologous systems.

## Graphical abstract

This study presents a vascularized 3D tumor model to investigate immune–stromal–tumor interactions under allogeneic PBMC perfusion. While immune cells induced tumor shrinkage, they also promoted vascular regression, stromal activation, and cancer cell invasion. These findings underscore the dual effects of immune activity and highlight the need for physiologically relevant in vitro platforms.

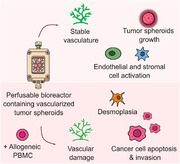

**Abbreviations**
CAF: cancer‐associated fibroblast
DCIS: ductal carcinoma in situ
ECM: extracellular matrix
FAP: fibroblast activation protein
HCC1937: human breast cancer cell line HCC1937
HUVEC: human umbilical vein endothelial cells
iPSC: induced pluripotent stem cell
MHC: major histocompatibility complex
nHDF: normal human dermal fibroblasts
PBMC: peripheral blood mononuclear cell
SHG: second harmonic generation
TNBC: triple‐negative breast cancer

## 1 Introduction

Animal experimentation remains essential for studying the immune tumor microenvironment. However, murine models inherently differ from humans, and tumor xenografts in humanized mice often fail to fully recapitulate human immune responses [1, 2]. Humanized mouse models, created by engrafting human hematopoietic stem cells into highly immunodeficient mice (e.g., NOD scid gamma mice), represent a step forward, yet their utility is limited by graft‐versus‐host reactions and impaired generation of antigen‐specific antibodies [3]. An alternative approach involves human‐mouse chimeras, in which human cells are integrated into multiple murine tissues. While these models can address some limitations of traditional xenograft mice, they also raise ethical concerns [2, 4, 5]. Given these challenges and the increasing pressures to adhere to the 3R principles “replacement, reduction, and refinement”, there is a growing need to complement in vivo models with 3D advanced all‐human in vitro systems [6]. These platforms provide a controlled environment to study immune cell trafficking and the dynamic interplay between immune, stromal, and tumor compartments [7]. Recent developments in all‐human 3D tumor models have enabled the integration of perfused vasculature to sustain cancer cell growth in microfluidic or bioreactor platforms. Here, tumor spheroids embedded in cell‐laden hydrogels support vascular network formation and matrix remodeling [8, 9, 10]. In addition to tumor, stromal, and endothelial cells, some of these systems now incorporate immune cells to more faithfully capture the complexity of the tumor microenvironment and to enable the study of immune‐tumor interactions. Typically, single immune cell populations, such as monocytes or NK cells derived from human peripheral blood mononuclear cells (PBMC), are introduced into the microfluidic devices a few days after establishing perfusable vascular networks [11, 12, 13]. These models have shown, for instance, that tumor‐associated macrophages within spheroids enhance monocyte infiltration and extravasation into the hydrogel, while monocyte presence in vascular structures can inhibit tumor cell extravasation [14, 15]. Beyond modeling immune trafficking, NK cells introduced into vascularized platforms on day 5 of culture were shown to have anti‐tumor effects, while the addition of genetically engineered CAR‐T cells targeting HER2 or CD19 resulted in tumor spheroid volume reduction and increased secretion of inflammatory cytokines [11, 12, 13].

While these studies assess immune efficacy through tumor spheroid shrinkage, this metric alone can overlook crucial cellular interactions within the tumor microenvironment, particularly between immune cells, tumor‐primed endothelial cells, and stromal elements, which are essential for a comprehensive understanding of anti‐tumor immune responses. However, introducing immune cells to a system comprised of cellular elements sourced from different donors creates allogeneic multi‐donor environments that can trigger incompatibility reactions reminiscent of graft‐versus‐host responses caused by major histocompatibility complex (MHC) mismatches. The risk of allogeneic immune activation may skew the interpretation of experimental outcomes and can limit the ability to use such models to reliably predict therapeutic efficacy [1].

Despite this, the impact of cellular incompatibility on stromal and tumor responses in such multi‐donor models is often underestimated and remains poorly understood. Although these in vitro platforms are not designed to replicate organ transplantation, the principle of combining allogeneic cellular components without immunosuppression parallels certain aspects of transplant pathology driven by immune incompatibility. In clinical settings, such dysregulated immune responses not only contribute to graft rejection but also elevate the risk of malignancies in transplant recipients, underscoring the importance of how allogeneic interactions influence cellular and tissue dynamics [16, 17, 18]. Similarly, within allogeneic in vitro culture systems, the contained cells may undergo significant changes in response to immune cell activity, including alterations in the extracellular matrix (ECM), such as increased collagen deposition and the expression of inflammatory and activation markers. Thus, a closer investigation of allogeneic interactions in 3D in vitro models is essential, both to minimize confounding effects caused by immune incompatibility and to elucidate the mechanisms by which immune activation drives stromal remodeling.

To this end, we utilized our previously developed perfusable bioreactor platform with a bioprinted multilayered vessel‐like structure to grow vascularized triple‐negative breast cancer (TNBC) tumor spheroids. We have already demonstrated that dynamic culture conditions in this platform promote the formation of a mature and functional (i.e., perfusable) vascular network [19]. Subsequently, this system was used to support the growth of artificial tumors, their transition toward a metastatic phenotype, and their interactions with the vascularized ECM [20]. Here, we examined the impact of allogeneic immune cell interactions on stromal and cancer cells by perfusing bioreactors containing vascularized breast cancer tumor spheroids with PBMC isolated from female donor blood. Tumor responses to PBMC perfusion were evaluated by analyzing cancer cell proliferation, apoptosis, and invasive behavior, as well as assessing vascular remodeling and fibroblast‐mediated desmoplasia. Immune cell subpopulations within the PBMC fraction were profiled before and after perfusion to quantify immune activation. These observations highlight the role of tumor‐derived signals in shaping stromal‐immune interactions and promoting localized matrix remodeling in an allogeneic setting.

## 2 Results

### 2.1 PBMC Addition to Vascularized Tumor Spheroids Causes Pronounced Vascular Regression

**FIGURE 1:**
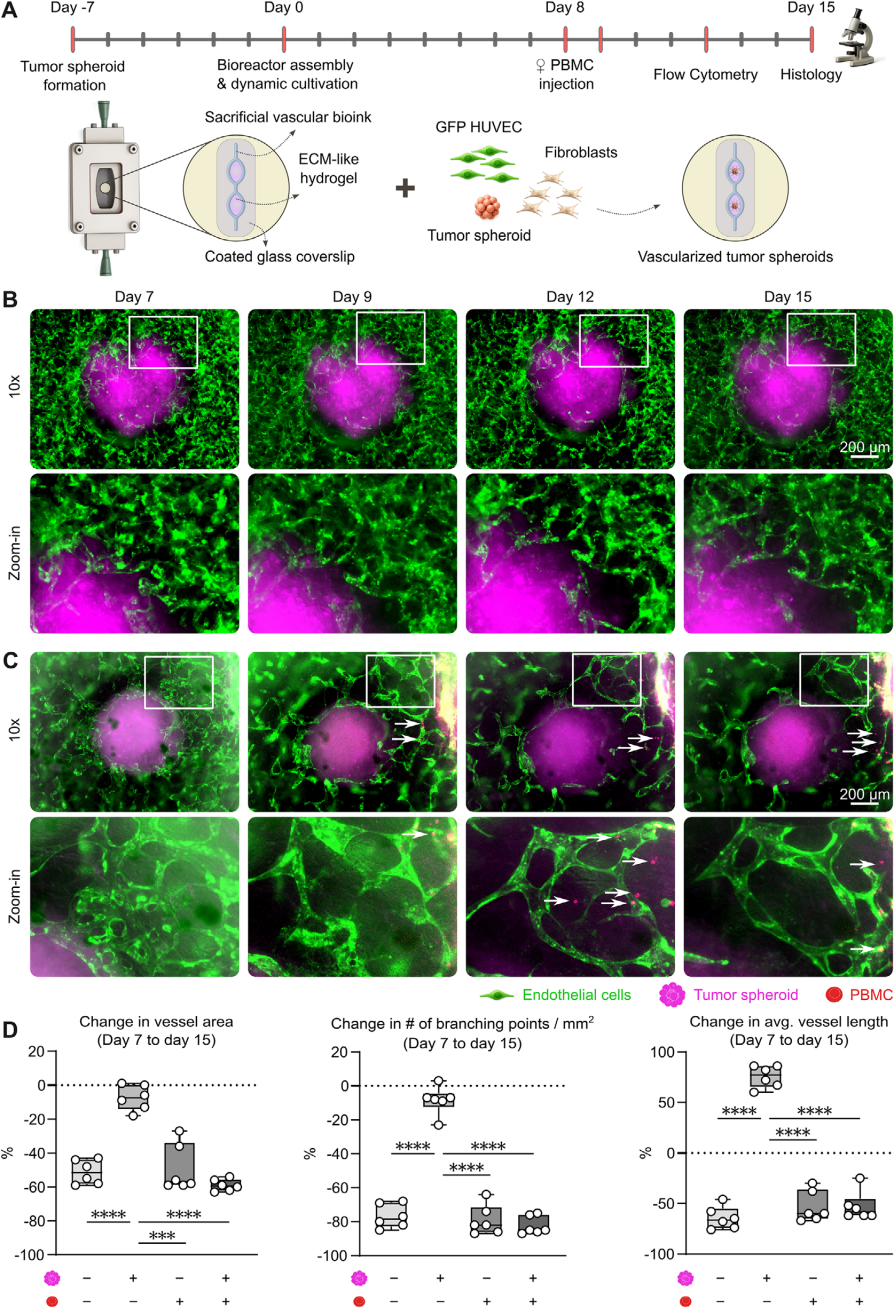
PBMC addition to a vascularized tumor model results in the depletion of vascular structures. (A) Timeline showing the experimental workflow: 7 days before the start of dynamic cultures, tumor spheroids were formed. After 8 days of dynamic cultivation, bioreactors were either perfused with or left without PBMC. Day 15 marks the experimental endpoint, when hydrogels were extracted to perform histological analysis. The schematic positioned above the timeline summarizes the bioreactor assembly process at day 0, including the embedding of cellular components into the hydrogel within defined geometries, as well as the resulting development of vascularized tumor spheroids. (Rendered with Illustrae.co) (B) After 7 days of cultivation, bioreactors feature vascularized tumor spheroids surrounded by a perfusable vascular network. From day 7 to day 15, the vasculature continued to develop and undergo dynamic remodeling. Magnified views of the regions outlined by white boxes highlight the interface between tumor spheroids and the surrounding infiltrating vascular network. (C) Upon the introduction of DiD‐labeled PBMC on day 8, significant vascular remodeling is observed already within the first 24 h post‐injection, followed by progressive regression until day 15. Magnified views of the regions outlined by white boxes highlight the interface between tumor spheroids and the surrounding vascular network, illustrating numerous infiltrating PBMC (white arrows) within vessels or surrounding the tumor spheroid. (D) To assess whether tumor spheroids modulated the effect of PBMC perfusion, vascular remodeling was quantified in bioreactors with and without tumor spheroids. While vascular networks in tumor‐free systems declined similarly regardless of PBMC exposure, tumor spheroids stabilized vessels in the absence of PBMC but triggered rapid and extensive vascular regression upon PBMC perfusion, resulting in significant reductions in vessel area, interconnectivity, and length by day 15. Data are displayed as mean ± SD of n = 6 bioreactors per group. Statistical significance was assessed using a Kruskal‐Wallis one‐way ANOVA test followed by Dunn´s post hoc correction test. P‐values: *** <0.001, **** <0.0001.

Using previously established methods for generating a long‐term stable mesoscopic tumor model with functional vasculature, we wanted to investigate the sequence by which an initially avascular tumor lesion becomes vascularized and subsequently, encounters circulating immune cells [1, 2]. To this end, TNBC tumor spheroids comprising HCC1937 cancer cells and adult dermal fibroblasts were embedded within a hydrogel matrix containing stromal and endothelial cells, surrounded by a bioengineered vascular network in a mesoscopic and imaging‐compatible bioreactor (Figure 1A) (Figure S1). Medium was perfused through the bioreactors at a flow rate of 0.1 mL min^−1^ through a single branched channel (diameter ≈ 1.5 mm). Based on previous ANSYS CFX simulations, this corresponds to a maximum flow velocity of approximately 4 mm s^−^^1^ and wall shear stress values around 5 mPa (0.05 dyn cm^−^^2^), which are sufficient for nutrient exchange, but remain far below thresholds associated with mechanical immune activation [20]. More specifically, reports in literature indicate Piezo1‐dependent immune cell mechanostimulation to occur at ≥ 50 mPa, placing shear levels in our system approximately one order of magnitude lower [21, 22, 23, 24].

Vascular structures were allowed to self‐assemble and mature until day 7. As shown in our previous work, this time point marks the onset of tumor spheroid vascularization. Additionally, our previous work verified the presence of functional and perfusable structures, demonstrated by the successful passage of fluorescent microbeads and red blood cells through interconnected lumina within the vascular network structures [20]. On day 8, bioreactors were either left perfused with control media (Figure 1A,B) or perfused with media where DiD‐labeled PBMC were resuspended (Figure 1A,C).

Shortly after PBMC perfusion, immune cells were observed adhering to the endothelium‐like structure and extravasating into the hydrogel. Following entry into the hydrogel, PBMC localized predominantly to areas adjacent to the vessel they had traversed and were also observed infiltrating the interstitial space between the tumor spheroid and the vessel. At later time points, PBMC were additionally detected within the tumor spheroid (Figure S2A,B).

To assess whether tumor spheroids influenced PBMC‐induced vascular regression, an additional experimental group without tumor spheroids was introduced. In both tumor‐free bioreactor conditions, with and without PBMC perfusion, vascular networks showed progressive regression. As previously reported, the vascular network in tumor‐free controls without PBMC was inherently less stable and showed early deterioration after day 7, with vessel density decreasing by 51% ± 6.47% between day 7 and 15 (Figure 1D) [20]. PBMC perfusion in the absence of tumor spheroids produced a comparable outcome, with reductions of 49% ± 12.80% in vessel density, 79% ± 7.97% in branching points, and 53% ± 13.11% in average vessel length by day 15 (Figure 1D). These findings indicate that PBMC exposure alone did not significantly impact vascular regression in the absence of tumor spheroids, and that both conditions ultimately resulted in a similarly degenerated vascular state characterized by reduced complexity and branching. In contrast, control bioreactors without PBMC exposure displayed stable or progressively growing vessel structures from day 7 to day 15, maintaining consistent spheroid perfusion throughout the experiment. (Figure 1C,D). Quantitative analysis revealed only minor reductions in vessel area 8% ± 6.75% and branching points 9% ± 7.61%, accompanied by a substantial increase in average vessel length by 76% ± 9.90%, indicative of active vascular remodeling and stabilization (Figure 1D). Remarkably, PBMC trafficking through the vascular network in tumor‐containing bioreactors was associated with rapid and dramatic vascular regression within 24 h post‐perfusion, characterized by a significant reduction in vessel density (Figure 1C). Vascular deterioration continued until day 15, ultimately resulting in the loss of most vessels surrounding the tumor spheroids. Importantly, regions of pronounced endothelial cell loss overlapped with hotspots of PBMC accumulation (Figure S2). More concretely, we quantified a reduction in the total vessel area of 59% ± 3.16%, a loss of vessel interconnectivity of 82% ± 4.89%, and an average vessel length reduction of 53% ± 14.10% (Figure 1C,D). These findings demonstrate that tumor spheroids, while initially stabilizing the vascular network, rendered the system highly susceptible to immune‐mediated damage upon PBMC exposure.

### 2.2 PBMC‐Induced Inflammation in the Tumor Microenvironment Causes Cancer Cell Apoptosis, but Promotes an Invasive Phenotype

**FIGURE 2:**
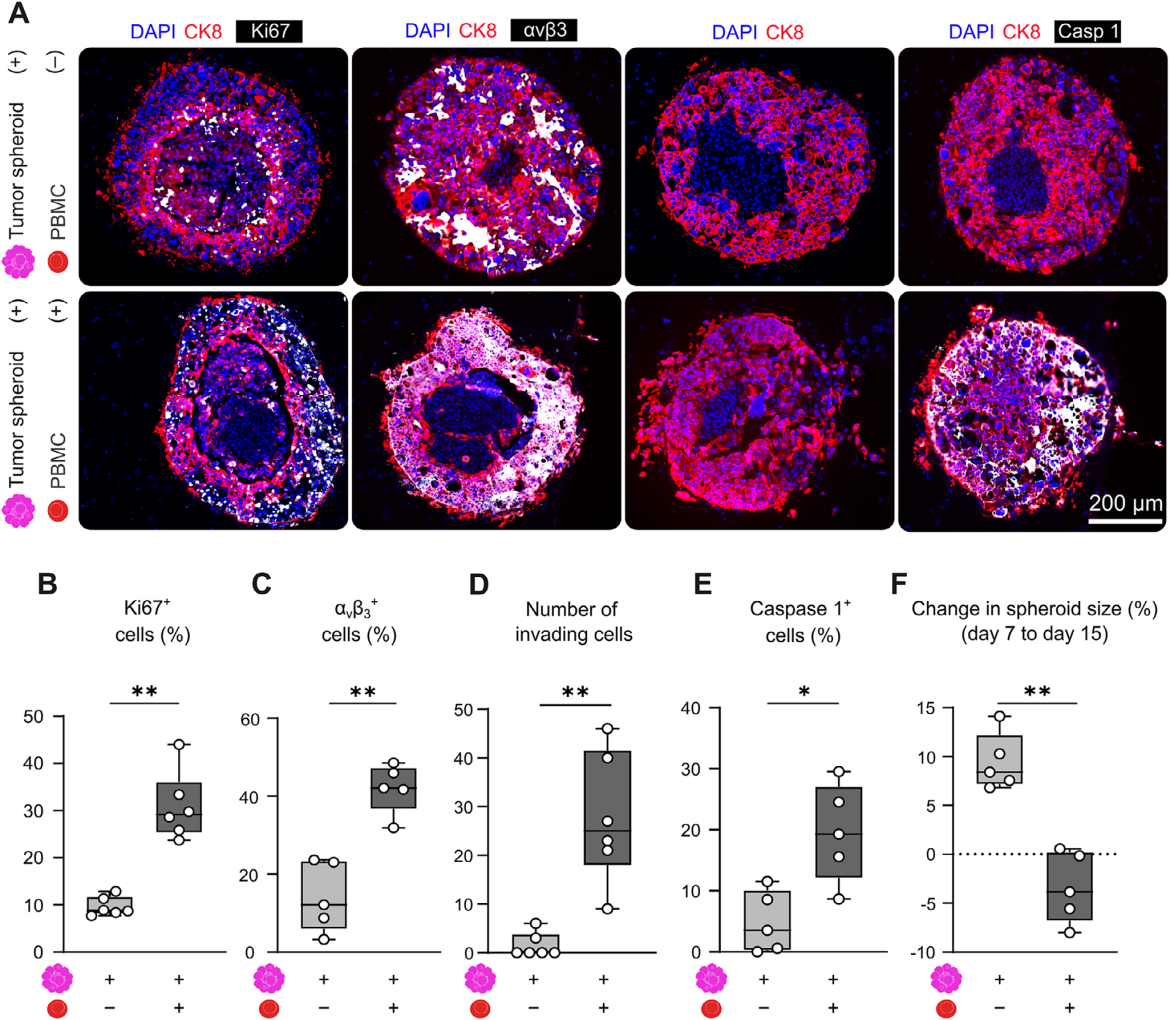
PBMC‐mediated inflammation induces cancer cell apoptosis and triggers invasive transition at the tumor‐stroma interface. (A) Representative fluorescence microscopy images show cross‐sections of tumor spheroids after 15 days of dynamic cultivation in bioreactors, comparing PBMC‐perfused and non‐perfused bioreactors. Images exemplify an increasing number of cells positively marked for Ki67, αvβ3, and caspase 1, and an increased amount of migrating tumor cells in PBMC‐administered bioreactors. (B–E) Quantification of immunofluorescence staining confirmed elevated levels of all analyzed markers following PBMC perfusion. Each of the analyzed markers was co‐stained with CK8 to distinguish cancer cells from fibroblasts. Ki67 (B) and αvβ3 integrin (C) levels in tumor cells increased, indicating enhanced proliferation and adhesion under inflammatory conditions. (D) An increased number of disseminated CK8^+^ tumor cells was observed migrating away from the spheroids, indicating a shift toward a more invasive tumor phenotype. Cancer cell invasion was quantified by counting CK8^+^ cells located at distances between 15 and 215 µm from the spheroid boundary, ensuring that only cells clearly separated from the main tumor mass were included in the analysis. (E) Caspase 1 levels increased, reflecting immune‐mediated cytotoxicity. (F) In line with these changes, overall tumor spheroid size decreased between day 7 and day 15 after PBMC introduction. Data are displayed as mean ± SD of n = 5‐6 spheroids per group. Statistical significance was assessed using a two‐tailed Mann‐Whitney test. P‐values: * <0.05, ** <0.01.

We next analyzed the effects of PBMC perfusion on the tumor spheroids, focusing on the expression of Ki67, α_v_β_3_ integrin, CK8, and caspase 1 (Figure 2A). In the absence of PBMC, vascularized tumor spheroids displayed homogeneously distributed Ki67^+^ cells, reflecting sustained cancer cell proliferation (Figure 2B). Upon PBMC perfusion, the number of Ki67^+^ cells significantly increased and localized predominantly to the outer rim of the spheroids, forming a dense proliferative zone. This spatial shift likely reflects a tumor cell response to immune‐mediated inflammation at the spheroid periphery.

The amount of α_v_β_3_ integrin^+^ tumor cells also increased following PBMC introduction (Figure 2C), suggesting enhanced adhesive and potentially invasive behavior of tumor cells under inflammatory conditions. Consistent with this, cancer cells, specifically identified by positive CK8 staining, adopted a more invasive phenotype, with both individual cells and small clusters infiltrating the surrounding microenvironment. On average, 28 ± 12 disseminated CK8^+^ cancer cells per spheroid were detected in PBMC‐perfused bioreactors, located at a distance between 15 and 215 µm from the spheroid body. Notably, this invasive behavior was absent in spheroids cultivated in PBMC‐free bioreactors, where tumor spheroids retained a more cohesive morphology and, on average 1.5 ± 2 CK8^+^ cancer cells invaded into the surrounding matrix (Figure 2D).

Apoptotic activity within tumor spheroids was also elevated in the presence of PBMC. Specifically, there was a marked increase in the amount of caspase 1^+^ cancer cells (Figure 2E), confirming immune‐mediated pyroptotic cell death and highlighting the contribution of inflammation‐induced cytotoxicity in modulating the tumor microenvironment. In addition, caspase 9 expression was significantly increased, with more than half of the cancer cells testing positive within PBMC‐perfused systems (Figure S3). The tumor microenvironmental changes induced by the introduction of PBMC, including loss of vascular patency and an increase in immune‐cell‐mediated cell death, ultimately led to changes in tumor spheroid size (Figure 2F). In PBMC‐free bioreactors, spheroids continued to grow between days 7 and 15, consistent with sustained proliferation indicated by Ki67 levels. In contrast, spheroids exposed to PBMC either remained the same size or shrank over time. This decline in spheroid area is consistent with increased apoptosis and pyroptosis outpacing proliferative expansion.

### 2.3 Cancer Cells Activate Endothelial Cells, Enhancing Their Interaction With PBMC

**FIGURE 3:**
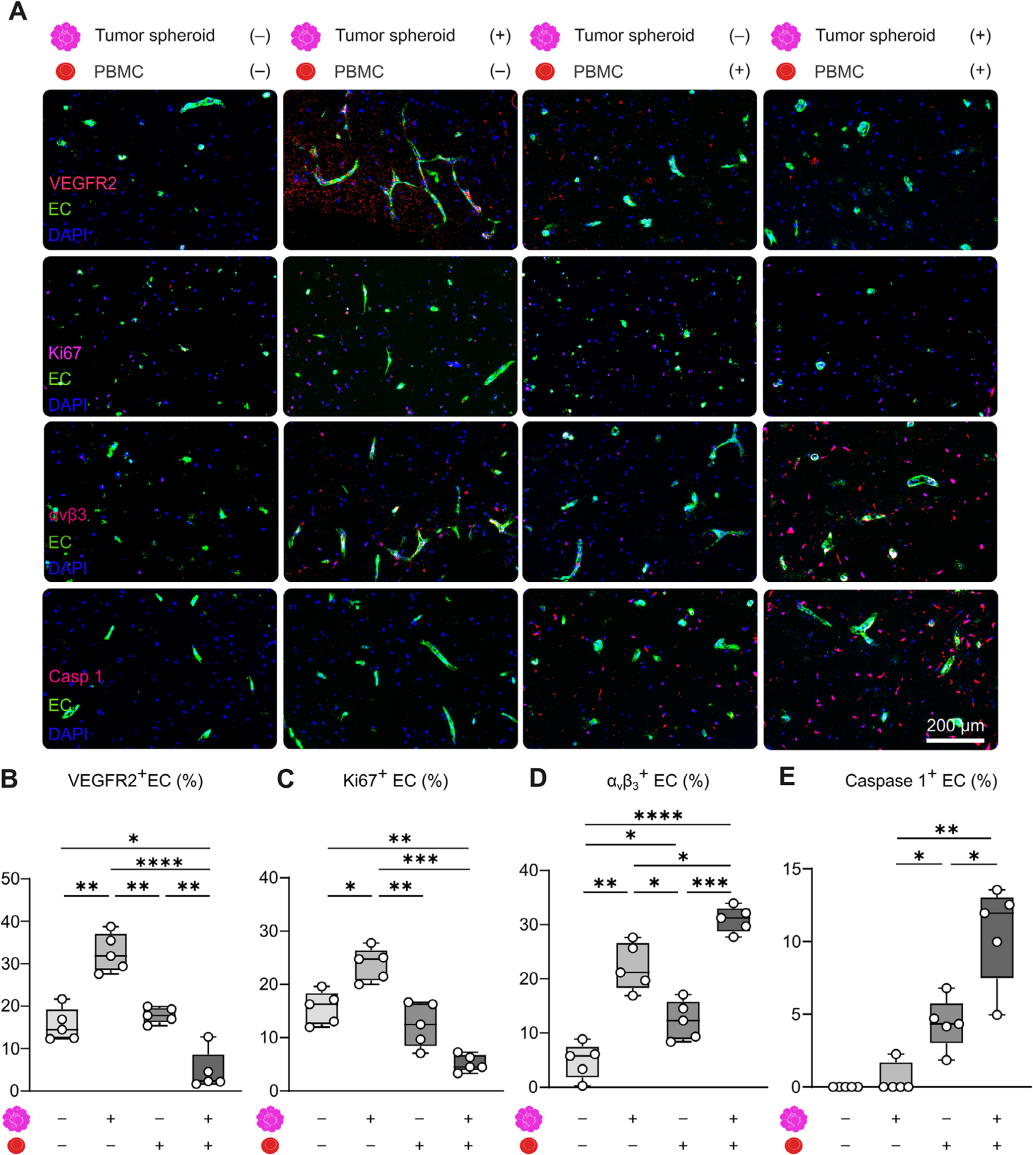
PBMC exposure suppresses endothelial growth and promotes caspase‐dependent endothelial damage. (A) Representative fluorescence microscopy images display the space surrounding tumor spheroids after 15 days of dynamic cultivation, showcasing reduced VEGFR2^+^ and Ki67^+^ cell counts, alongside elevated levels of α_v_β_3_ integrin and caspase 1 upon PBMC perfusion in the bioreactors. (B–D) Quantification of immunofluorescence staining reveals a significantly higher amount of cells that express endothelial activation markers VEGFR2 (B), Ki67 (C), and αvβ3 integrin (D) in bioreactors containing tumor spheroids compared to those without. PBMC perfusion reduces endothelial proliferation and angiogenic signaling (VEGFR2, Ki67), while increased αvβ3 integrin expression reflects an enhanced inflammatory response. (E) Increased Caspase 1 levels following PBMC introduction confirmed immune‐mediated endothelial cell death. Data are displayed as mean ± SD of n = 5 bioreactors per group. Statistical significance was assessed using a Kruskal‐Wallis ANOVA test followed by Dunn´s multiple comparison correction. P‐values: * <0.05, ** <0.01, *** <0.001, **** <0.0001.

To assess immune‐endothelial cell interactions, we performed histological analyses focusing on vascular activation, proliferation, and cell death (Figure 3A–E). Angiogenic and proliferative responses were evaluated using VEGFR2 and Ki67. Endothelial cells in bioreactors containing tumor spheroids showed significantly higher expression of both markers compared to tumor‐free controls, confirming tumor‐induced endothelial activation. In tumor‐free bioreactors, PBMC perfusion had minimal impact on VEGFR2 and Ki67 expression. However, in the presence of tumor spheroids, PBMC addition led to a marked reduction in both markers, suggesting a loss of angiogenic and proliferative capacity (Figure 3B,C). These findings indicate that tumor‐primed endothelial cells are more susceptible to immune‐mediated dysfunction.

We next analyzed α_v_β_3_ integrin, a marker of vascular activation. Similar to VEGFR2 and Ki67, α_v_β_3_ integrin expression was elevated in tumor‐containing bioreactors, consistent with an activated endothelial phenotype (Figure 3D). PBMC perfusion further increased α_v_β_3_ levels in both tumor‐free and tumor‐containing systems, with a more pronounced effect in the latter. These findings suggest that the PBMC‐induced inflammation is pronounced when tumor spheroids are present in the hydrogel, further emphasizing the role of tumor‐primed endothelial cells in shaping immune responses. To understand the mechanisms underlying PBMC‐induced vascular regression, we examined endothelial cell death pathways. Caspase 9, associated with intrinsic (mitochondrial) apoptosis, was minimally expressed in tumor‐containing systems but slightly elevated in tumor‐free bioreactors, likely reflecting baseline vascular fragility (Figure S4). PBMC perfusion increased the number of caspase 9^+^ cells in both conditions, especially in tumor‐primed vessels. Additionally, caspase 1, a key effector of the inflammasome pathway associated with pyroptosis, was strongly upregulated following PBMC exposure, confirming immune‐mediated endothelial cell damage (Figure 3E). Notably, tumor‐activated endothelial cells exhibited greater caspase 1 induction than their non‐activated counterparts, underscoring their heightened vulnerability to immune‐mediated cytotoxicity.

### 2.4 PBMC Induce a Desmoplastic Reaction in Tumor‐Activated Fibroblasts

**FIGURE 4:**
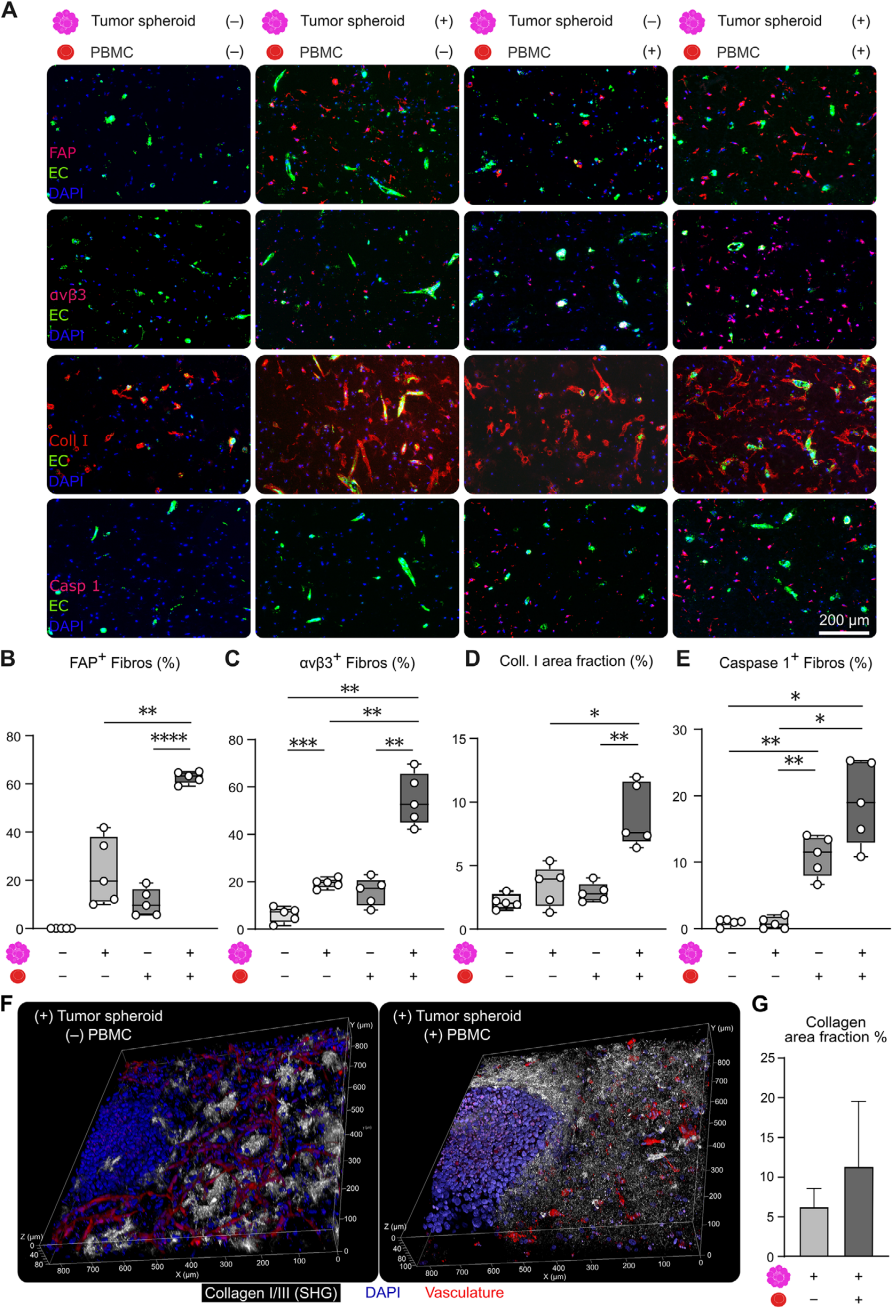
PBMC‐driven inflammation amplifies fibroblast activation and desmoplasia, accompanied by pyroptotic cell death. (A) Representative fluorescence microscopy images display the space surrounding tumor spheroids after 15 days of dynamic cultivation and show increased amounts of FAP^+^ cells and collagen I deposition in the tumor microenvironment following PBMC perfusion. (B–E) Quantification of immunofluorescence staining revealed elevated levels of fibroblast activation markers αvβ3 integrin (B) and FAP (C) in bioreactors with tumor spheroids compared to those without. PBMC addition significantly increases both FAP and αvβ3 integrin expression, highlighting enhanced inflammatory responses. This is further supported by increased collagen I deposition following PBMC perfusion (D). (E) Statistically significant increase in caspase 1^+^ fibroblasts upon PBMC perfusion specifically confirms immune‐mediated fibroblast cell death. (F) Two‐photon laser scanning microscopy with second harmonic generation (SHG) visualization of fibrillar Collagen I/III. In bioreactors without PBMC perfusion (left), collagen appears in spatially restricted hotspots dispersed throughout the hydrogel matrix. In contrast, PBMC‐perfused bioreactors (right) display an increased and more homogenously distributed collagen signal, including a dense collagen accumulation in proximity to the tumor spheroid. (G) Quantification of the SHG‐derived Collagen I/III area fraction in maximum intensity projection images of the entire sample area. PBMC‐perfused bioreactors show an increase in collagen area fraction compared to non‐perfused controls, indicating enhanced matrix deposition and remodeling following PBMC exposure. Data in (B‐E) are displayed as mean ± SD of n = 5 bioreactors per group. Statistical significance was assessed using a Kruskal‐Wallis ANOVA test followed by Dunn´s post hoc correction. P‐values: * <0.05, ** <0.01, *** <0.001, **** <0.0001. Data in (G) are displayed as mean ± SD of n = 3 bioreactors per group.

In addition to the vascular analysis, we examined immune–fibroblast interactions, as fibroblasts are key modulators of inflammation in the tumor microenvironment. Fibroblast activation was assessed using α_v_β_3_ integrin, Ki67, and fibroblast activation protein (FAP), a well‐established marker of cancer‐associated fibroblasts (CAF) (Figure 4A). Fibroblasts in bioreactors containing tumor spheroids exhibited elevated expression of all three markers compared to tumor‐free conditions, confirming activation by cancer cells.

PBMC perfusion further enhanced α_v_β_3_ integrin and FAP expression in tumor‐primed fibroblasts, whereas fibroblasts in tumor‐free bioreactors showed only modest increases (Figure 4B,C). Like in endothelial cells, numbers of Ki67^+^ fibroblasts decreased following PBMC addition, indicating reduced proliferative capacity despite increased activation (Figure S4). These findings highlight a consistent pattern across stromal cell types, where PBMCs escalate inflammation while dampening proliferation in cells pre‐activated by the tumor.

Furthermore, we studied collagen I deposition as an indicator of fibroblast response to inflammation (Figure 4D). In tumor‐free bioreactors, PBMC perfusion only led to a marginal increase in collagen I. Interestingly, tumor‐activated fibroblasts showed a marked elevation in collagen I levels following PBMC exposure, suggesting an enhanced response to immune‐mediated cues (Figure 4D). A more in‐depth analysis of fibrillar collagen revealed that in tumor‐containing bioreactors without PBMC, collagen I/III appeared organized in discrete hotspots, whereas PBMC perfusion resulted in a widespread and interconnected collagen network that was less clustered locally, but more uniformly distributed throughout the matrix (Figure 4F). Consistently, area fraction analysis showed elevated fibrillar collagen coverage after PBMC perfusion (Figure 4G).

Trends in apoptosis markers, caspase 9 and caspase 1, aligned with previous findings in endothelial cells. Caspase 9 levels were lowest in fibroblasts from tumor‐containing bioreactors without PBMC but increased sharply upon PBMC addition, particularly in tumor‐activated fibroblasts (Figure S4). Caspase 1 showed the strongest upregulation in tumor‐primed fibroblasts found in PBMC‐perfused systems (Figure 4E). These results confirm that tumor‐activated fibroblasts and tumor‐activated endothelial cells are more susceptible to immune‐mediated cytotoxicity, collectively contributing to a more reactive, fibrotic, and pro‐inflammatory stromal environment that may promote tissue dysfunction and tumor progression.

### 2.5 Tumor Spheroid‐Containing Bioreactors Induce Myeloid Persistence and Innate Cytokine Signaling

**FIGURE 5:**
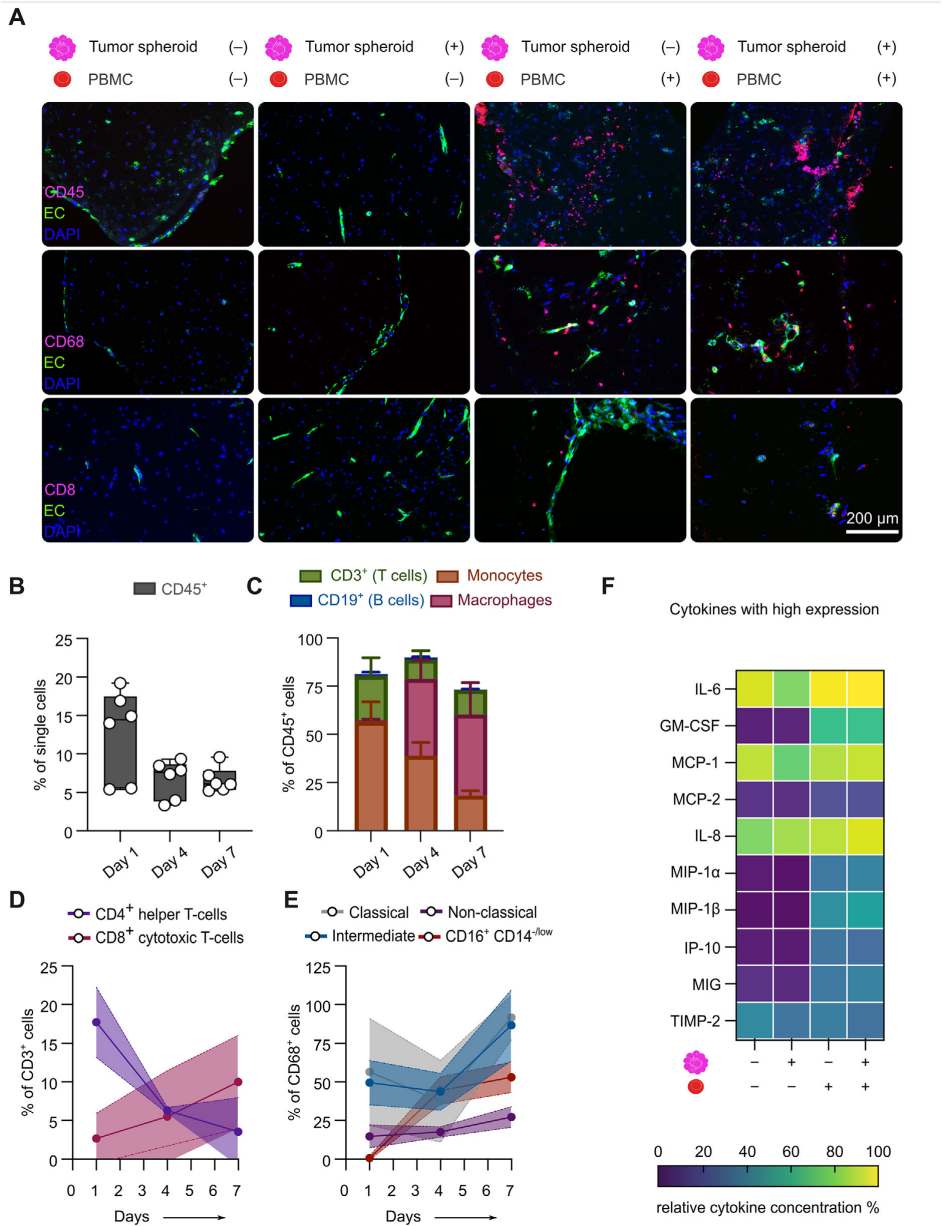
Myeloid populations dominate over cytotoxic T cells and drive innate inflammatory cytokine responses in PBMC‐perfused bioreactors. (A) Representative fluorescence microscopy images display the space surrounding tumor spheroids or cell‐laden hydrogels lacking tumor spheroids after 15 days of cultivation, confirming the presence of CD45^+^, CD68^+^, and CD8^+^ immune cells. (B–E) Flow‐cytometry analysis of CD45^+^ immune‐cell engraftment within the tumor microenvironment from day 1 to day 7 after PBMC perfusion. Quantification of CD45^+^ leukocytes as a fraction of all single cells recovered after enzymatic digestion reveals an initial decline in engraftment between day 1 and day 4, followed by a stable plateau that persists through endpoint analysis (B). Profiling of the CD45^+^ compartment demonstrates a contraction of T cell, B cell, and monocyte populations by day 4, whereas macrophage frequencies increase, reflecting a shift toward myeloid enrichment (C). Flow‐cytometry analysis of CD3^+^ T cell subsets shows a decrease in CD4^+^ helper T cells (pink) and a concomitant increase in CD8^+^ cytotoxic T cells (purple) (D). CD68 expression across classical (CD14^+^^+^CD16^−^, grey), intermediate (CD14^+^^+^CD16^+^, blue), non‐classical (CD14^+^CD16^+^^+^, purple), and CD16^+^CD14^−^/low (dark red) monocyte subsets on days 1, 4, and 7 post perfusion (E). The percentages of CD68^+^ cells within each subset indicate progressive differentiation toward a macrophage‐like phenotype. Data are presented as mean ± SD (n = 3 bioreactors). (F) Relative inflammatory cytokine concentrations (%) in the culture medium of the four different conditions. Media were pooled from n = 3 independent experiments with n = 4 bioreactors per experiment and condition after 15 days of cultivation. Inflammatory cytokines with a high expression profile show an increased expression of cytokines associated with monocyte activation and macrophage recruitment (concentrations range from 0%–100%).

To assess immune cell persistence and composition within the bioreactor systems following 15 days of dynamic culture, we performed immunofluorescent staining for the immune cell markers CD45 (pan‐leucocyte marker), CD68 (pan‐monocyte/macrophage marker), and CD8 (cytotoxic T cells) (Figure 5A). CD45^+^ cells, as well as CD68^+^ and CD8^+^ cells, were consistently detected in PBMC‐containing conditions with and without tumor spheroids at the experimental endpoint, confirming both myeloid and lymphoid immune cell engraftment. Notably, CD68^+^ macrophages were more abundant than CD8^+^ cytotoxic T cells, suggesting a dominant myeloid presence within the TME.

To analyze the dynamics of immune cell subpopulations and engraftment within the hydrogel over time, we performed flow cytometry analysis at days 1, 4, and 7 after PBMC addition, corresponding to the microscopy time points (Figure 1B,C). PBMC were also analyzed before bioreactor perfusion to provide an overview of the cellular composition, showing expected immune cell subset compositions [25]. Gating strategies are detailed in the Supplementary Information (Figure S5A, B). Flow cytometry enabled quantitative tracking of PBMC engraftment and revealed changes in absolute CD45^+^ immune‐cell numbers recovered from the tumor‐containing bioreactors over time (n = 3 bioreactors per time point): 982 ± 392 cells on day 1, 1297 ± 532 on day 4, and 239 ± 57 on day 7 post PBMC injection. The reduction in the number of recovered cells at later time points likely reflects increased ECM deposition, triggered by immune cell infiltration, that impairs efficient enzymatic niche dissociation. However, CD45^+^ cells as a percentage of all single cells isolated from the hydrogel stabilized after day 4 post PBMC injection with 6.73 ± 2.26% and 6.61 ± 1.48% on day 7, indicating sustained and robust immune cell engraftment throughout later stages of the experiment (Figure 5B).

Moreover, distinct temporal shifts were observed within both lymphoid and myeloid subpopulations. CD19^+^ B cells, which initially represented 11.20% of the CD45^+^ compartment (Figure S5A), declined rapidly after perfusion, dropping to 0.90 ± 0.76% by day 1 and further decreasing to 0.12 ± 0.17% by day 7 (Figure 5C; Figure S5C). CD3^+^ T cells similarly contracted over time, decreasing from 36.90% at baseline to 23.03 ± 7.63% on day 1 and stabilizing at 12.83 ± 2.93% by day 7 (Figure 5C; Figure S5C). Within the CD3^+^ compartment, CD4^+^ T helper cells declined markedly, suggesting limited survival or retention in the hydrogel niche. In contrast, CD8^+^ cytotoxic T cells increased as a relative proportion of the diminishing CD3^+^ pool, rising from 2.68 ± 2.66% on day 1 to 10.02 ± 4.94% by the experimental endpoint (Figure 5D). These dynamics indicate a selective persistence or enrichment of cytotoxic T cell subsets within the hydrogel over time. Myeloid subtypes revealed a strong shift toward macrophage activation. While CD68^+^ macrophages were absent before perfusion, 21% of the initial PBMC population were monocytes. Post‐engraftment, monocytes declined from 56.56 ± 8.39% (day 1) to 18.34 ± 2.02% (day 7), while CD68^+^ macrophages increased from 0.82 ± 0.49% to 42.23 ± 13.43% on the respective days (Figure 5E). Further subtyping of monocytes demonstrated a progressive increase in CD68 expression across all subsets, consistent with monocyte activation. By day 7, intermediate monocytes showed 80 ± 20% CD68 positivity, non‐classical monocytes reached 91.67 ± 11.79%, and CD16^+^CD14^‐low^ monocytes increased significantly from day 1 post PBMC injection to day 7 with 53.1 ± 1.13% CD68 expression (Figure 5E). Classical monocytes maintained lower activation levels of 27.3 ± 5.51% at the experimental endpoint. These trends support a myeloid shift dominated by macrophage differentiation and highlight robust and sustained macrophage engraftment and activation within the tumor niche, reinforcing their central role in shaping the immune landscape in this model.

To further characterize immune activity across conditions, we profiled the expression of inflammatory cytokines secreted in the media (Figure 5F and Figure S6). IL‐6, IL‐8, and MCP‐1 were among the most abundantly expressed cytokines. Of note, their expression level was already high in the absence of PBMC and tumor spheroids and increased further upon PBMC addition (Figure 5F). The elevated baseline secretion of IL‐6, IL‐8, and MCP‐1 may reflect stress‐induced stimulation of pro‐inflammatory pathways, such as NF‐κB, potentially resulting from the hydrogel's composition and stiffness, or by hypoxic conditions developing throughout prolonged dynamic culture [26]. However, follow‐up immunofluorescence staining for HIF‐1α revealed no detectable differences between conditions, suggesting that hypoxia is unlikely to be a major contributor to the observed cytokine patterns within our setup.

Additionally, several cytokines such as GM‐CSF, MIP‐1α, MIP‐1β, and MCP‐2, known mediators of monocyte activation and macrophage recruitment, were selectively upregulated in PBMC‐containing conditions, suggesting immune‐specific activation. Relative GM‐CSF levels increased to an average of around 66% in PBMC conditions, implicating immune‐derived signaling likely involved in promoting the expansion and differentiation of infiltrating myeloid cells. Notably, the expression of MIP‐1α and MIP‐1β, both secreted by activated macrophages, was higher in bioreactors containing both tumor spheroids and PBMC compared to systems where PBMC were introduced into tumor‐lacking bioreactors. This suggests that tumor‐derived cues further amplify myeloid cell recruitment and activation, consistent with the elevated presence of CD68^+^ cells observed in (Figure 5A,C). Together, these patterns of cytokine expression reflect a myeloid‐dominated immune profile, in which monocyte‐derived cells likely act as key mediators of vascular and ECM remodeling, as well as broader immune activation.

The expression of other inflammatory cytokines remained relatively low across all groups (Figure S7). Notably, IL‐2, IL‐17, IFN‐γ, and TNF‐α, cytokines typically associated with T cell activation, were either undetectable or expressed at minimal levels. The overall cytokine profile supports a shift toward an innate‐like response, characterized by monocyte recruitment and stromal‐immune crosstalk, rather than coordinated cytotoxic T cell engagement.

Finally, to determine which of the cytokines elevated in the circulating medium during PBMC perfusion contribute to tumor cell migration and invasion, we performed complementary 2D and 3D invasion assays (Figure S7). Through this screening, we have identified that MCP‐1 and MIP‐1α promoted migration across multiple concentrations in 2D. In addition, IL‐6‐containing cytokine combinations most strongly enhanced wound closure in 2D and tumor cell dissemination in 3D. These findings support the notion that the cytokines most elevated under PBMC perfusion, particularly IL‐6, serve as key mediators linking inflammatory activation to tumor cell invasiveness.

## 3 Discussion

Most in vitro tumor platforms rely on cellular components sourced from different or pooled donors [11, 12, 13, 15]. While such approaches can simplify experimental design, they introduce variability and potential confounding effects that may significantly influence immune responses and therapeutic outcomes. To develop robust in vitro tumor models for studying immunotherapeutic drugs and immune cell behavior, it is essential to understand how donor mismatch and the integration of heterogeneous cell sources shape stromal activation, immune infiltration, and tumor remodeling. To this end, we established a vascularized tumor model incorporating tumor spheroids and multi‐donor endothelial cells, fibroblasts, and PBMC.

In our model, PBMC introduction led to an overall reduction in tumor spheroid size. This was associated with increased levels of both apoptosis and pyroptosis in PBMC‐exposed spheroids, likely contributing to the observed size decrease. This finding aligns with previous reports, which show that immune cell infiltration can induce tumor cell death and significantly reduce tumor size in vitro [11, 12, 13]. However, in addition to this cytotoxic response, we also observed increased cancer cell proliferation at the spheroid periphery, elevated numbers of α_v_β_3_ integrin^+^ cancer cells, and a significantly increased number of migrating cancer cells upon bioreactor perfusion with PBMC. These observations are consistent with the well‐established dual role of inflammation in cancer, where acute immune activation can suppress tumor growth, whereas chronic or unresolved inflammation may promote tumor progression, immune evasion, and cancer cell invasion [27].

Moreover, the cytokine profile induced by PBMC perfusion further supports the association between inflammation and cancer cell invasiveness. IL‐6, a key cytokine upregulated in PBMC‐perfused bioreactors, is known to activate JAK/STAT3 signaling, promoting epithelial‐to‐mesenchymal transition (EMT), cancer stemness, and α_v_β_3_ integrin expression, particularly in TNBC and other solid tumors [28, 29, 30]. We similarly found IL‐6 to be the key mediator of tumor cell invasion within 3D static invasion assays. IL‐8, which we found to also be strongly upregulated, can promote actin remodeling, chemotaxis, and EMT‐like phenotypes in TNBC via CXCR1/2 signaling, reinforcing a migratory and invasive tumor state [31]. Additionally, elevated levels of MCP‐1 (CCL2) and MIP‐1α (CCL3) chemokines, known to recruit monocytes and stimulate ECM remodeling, contribute to the establishment of a pro‐invasive niche, with CCL2 directly promoting EMT and invasion of TNBC cells [32]. These cytokine‐mediated interactions likely created a permissive microenvironment for tumor cell dissemination from the spheroid into the surrounding matrix [33].

Next to this, we observed increased amounts of FAP^+^ cells in the stromal compartment, indicating enhanced CAF activity in tumor‐containing and PBMC‐perfused bioreactors. This points toward a reinforcing loop of immune‐stroma‐tumor interaction, where inflammatory myeloid cells secrete cytokines that activate CAF and endothelial cells, which in turn remodel the extracellular matrix, whilst releasing additional chemokines [34]. As a result, tumor cells respond by upregulating invasive cues, mirroring in vivo observations, where chronic inflammation and desmoplasia combine to drive tumor progression [34]. This inflammatory remodeling also has important implications for immune surveillance. Persistent inflammation and ECM remodeling are known to restrict T cell infiltration, contributing to immune evasion and an immune‐excluded tumor phenotype [35]. In our model, this is reflected by elevated IL‐6 and IL‐8 levels alongside dominant myeloid infiltrates, both features that are commonly associated with poor prognosis and reduced immunotherapy responsiveness [35]. These inflammatory mediators can suppress antigen presentation and upregulate immune checkpoint molecules, further limiting cytotoxic T cell activity [36].

Our observation of reduced tumor size alongside increased invasiveness aligns with prior studies demonstrating that immune infiltration or chemotherapeutic interventions can induce tumor cell death, through processes like immunoediting, allowing more proliferative subpopulations to persist and expand [37, 38, 39]. In breast cancer, for example, adjuvant chemotherapy reduces tumor burden in hormone receptor‐negative subtypes, yet residual tumor cells often survive and drive relapse with more aggressive characteristics [40]. Moreover, therapy‐induced apoptosis has been shown to trigger compensatory proliferation in surviving cells through apoptosis‐induced proliferation, a process driven by caspase‐3 and prostaglandin E2 signaling [41].

Tumor spheroids also profoundly influenced the surrounding microenvironment by stabilizing the vasculature and promoting endothelial cell proliferation and angiogenic activity. This effect is consistent with the release of pro‐angiogenic factors by breast cancer cells, including VEGF and IL‐8, which stimulate endothelial outgrowth, vessel sprouting, and survival [42, 43, 44]. Notably, in ductal carcinoma in situ (DCIS), angiogenic signaling precedes tumor invasion and contributes to stromal activation. DCIS lesions frequently exhibit elevated VEGF expression within the ductal epithelium, which is linked to increased microvessel density at the tumor‐stroma interface and early CAF activation [45]. This VEGF‐driven signaling promotes vascular proliferation and stromal remodeling, including myofibroblast expansion and fibronectin deposition, thereby establishing a permissive niche for tumor progression [46].

Similarly, in our model, tumor‐derived signals activated fibroblasts and sensitized both stromal and endothelial compartments to subsequent inflammatory cues upon PBMC exposure. This resulted in a shift from pro‐angiogenic to inflammatory phenotypes, where endothelial cells exhibited reduced angiogenic capacity and upregulated inflammatory markers, while fibroblasts mounted a desmoplastic response marked by increased collagen deposition and an increase in FAP expression.

As major producers of collagen and other ECM components, CAFs contribute to the formation of a dense, fibrotic stroma that limits immune infiltration. In solid tumors, including TNBC and pancreatic cancer, CAF‐mediated matrix remodeling creates a physical, protective barrier that limits lymphocyte access to tumors [47]. The depletion of CAF in murine models, for instance, led to the collapse of the stroma and restored infiltration of cytotoxic T cells and immune checkpoint antibodies [48]. In TNBC, a distinct CAF subtype known as “ECM‐CAF” has been linked to T cell exclusion and fibrotic stromal expansion by physically trapping immune cells within the stroma and by downregulating endothelial adhesion molecules [47]. In line with this, high collagen density with reduced T cell infiltration is linked to poor response to immunotherapy, whereas targeting FAP‐positive CAF can disrupt the ECM barrier and reverse immune exclusion [49, 50].

Beyond immune exclusion, CAF can also influence tumor vascularization in context‐dependent ways. On one hand, they promote neovascularization by secreting pro‐angiogenic factors, particularly under hypoxic or oncogenic conditions [51, 52]. In BRCA1‐mutant TNBC, inflammatory CAF have been shown to engage endothelial tip cells to enhance new vessel sprouting directly [53]. Conversely, anti‐angiogenic CAF subsets have also been identified, such as those in hepatocellular carcinoma that inhibit vascular growth through the secretion of angiostatic factors [54, 55]. Similarly, 3D in vitro models of colorectal carcinoma have demonstrated CAF‐mediated suppression of vascular network formation, accompanied by elevated expression of factors known to restrict angiogenesis [56].

Furthermore, our system revealed a predominance of CD68^+^ myeloid cell accumulation over time. Although the original PBMC populations were enriched in CD8^+^ T cells, we observed only modest T cell infiltration and minimal adaptive cytokine expression at the experimental endpoint. The distribution of classical, intermediate, and non‐classical monocytes also shifted over time, reflecting a temporal shift toward more activated and macrophage‐like monocyte states. These subsets are each known to support key tumor‐associated functions, including recruitment, cytokine amplification, and differentiation into tumor‐associated macrophages. Notably, the observed progression from classical CD14^+^^+^ monocytes toward CD16^+^ intermediate and macrophage‐like CD68^+^ cells is consistent with well‐established monocyte to tumor‐associated‐macrophage differentiation pathways described in solid tumors [57, 58]. The limited T cell activity may not be mechanistically driven by the tumor spheroid alone but could also arise from the allogeneic nature of the system and insufficient cytokine support for sustained T cell survival. In addition to immune exclusion by desmoplastic cues, MHC I expression on tumor and stromal cells may further influence the extent of T cell engagement, as tumor‐associated downregulation of MHC I is a well‐known immune escape mechanism in solid cancers [59, 60, 61]. While the dense, collagen‐rich hydrogel matrix likely creates a physical and biochemical barrier that preferentially limits T cell infiltration compared to macrophages, other effects, such as T cell exhaustion or loss of viability under prolonged conditions without continuous cytokine signaling, must also be considered [62, 63]. Although mismatched donor combinations can provoke allogeneic immune responses, our system did not exhibit a graft‐versus‐host‐like reaction with robust T cell infiltration or T cell‐mediated cytotoxicity. Instead, it more closely reflected an early inflammatory or reparative niche dominated by innate immune dynamics and stromal remodeling [18].

One of the central limitations of current in vitro platforms, including our own, is precisely this reliance on unmatched donor components. While this multi‐donor configuration enables practical and scalable experimentation, it can introduce allogeneic interactions that influence stromal‐immune crosstalk and affect matrix remodeling, immune infiltration, and inflammatory signaling. With this work, we also aim to draw attention to the types of responses that may arise specifically in mixed‐donor systems, as these effects warrant caution, especially when using these models to evaluate immunotherapies. Despite these limitations, the processes captured in our model, including vascular regression, myeloid accumulation, stromal activation, and enhanced tumor cell invasion, closely mirror in vivo phenomena, suggesting that mechanisms uncovered with this platform remain biologically meaningful.

Nonetheless, the development of truly syngeneic, autologous models, in which all four components, including stromal, endothelial, tumor, and immune cells, are derived from a single patient, represents the next steps in translational in vitro tumor modeling. Recent work, in which authors successfully extracted all required cell types directly from patient lung tumor biopsies to seed a microfluidic platform, elegantly demonstrates the feasibility of autologous configurations and provides a valuable foundation for future studies [64]. However, generating patient‐matched endothelial and stromal cells in significant quantities and with functional maturity poses financial and time‐related challenges [65]. Similarly, the establishment and culture of patient‐derived tumor organoids is costly and can often be unpredictable in terms of success, particularly in pretreated patients, where prior therapies can compromise organoid viability and expansion ability [66, 67]. While induced pluripotent stem cell (iPSC)‐derived cells in combination with tumor organoids isolated from patient biopsies hold promise, no platform to date has successfully integrated all four components within a fully autologous format. However, vascularized tumor models based on patient‐derived cancer cell lines have already begun to capture some aspects of tumor heterogeneity and microenvironmental complexity [20, 68]. Furthermore, studies using patient‐derived colorectal cancer organoids have demonstrated distinct immune responses between autologous and allogeneic PBMC, with allogeneic PBMC causing increased tumor damage due to additional immune activation through alloreactivity [69]. Currently, we are actively working toward establishing such syngeneic models within our group, as we recognize that their realization will enable a more accurate understanding of immune‐cancer interplay, whilst ruling out donor mismatch artefacts. Importantly, the mesoscopic‐scale nature of our platform is well‐suited to accommodate larger patient‐derived tumor organoids and the integration of patient‐matched endothelial and stromal cells for the formation of vascular networks to supply the organoids. The incorporation of patient‐specific immune cells under flow would furthermore enable fully personalized tumor‐immune interaction studies and pave the way toward personalized drug screening applications. At the same time, further refinement and standardization of multi‐donor models, paired with careful control of donor mismatch, will remain essential for translational research, as these systems offer a cost‐efficient and experimentally accessible foundation that can be implemented, while autologous platforms continue to be developed.

## 4 Conclusion

In this study, we present an in vitro platform that integrates a vascularized tumor model with functional, perfused vasculature capable of modelling immune cell trafficking. We demonstrate that the introduction of allogeneic PBMC into the system led to a significant loss of vascular integrity due to the establishment of an inflammatory environment. The presence of tumor spheroids within the bioreactors is not only essential for maintaining vascular network stability, but also actively drives the activation of stromal and endothelial components. Therefore, tumor‐derived signaling further amplifies stromal‐immune interactions, contributing to pronounced matrix remodeling and inflammation in the tumor microenvironment with innate, wound‐healing‐like immune responses. Importantly, these observations were made in an allogeneic, multi‐donor setting, combining all cell types from different donors. While this approach captures the complexity of mixed immune cell infiltration, it also addresses potential donor mismatch effects, possibly influencing inflammation, as well as cellular and matrix remodeling within the presented tumor microenvironment. Notably, similar phenomena are observed clinically, where the introduction of allogeneic tissues, such as during organ transplantation, can provoke a more aggressive remodeling of the surrounding tissue environment. Additionally, resulting dysregulated immune activation can not only contribute to graft rejection, but may also be implicated as a bystander factor in tumor promotion, next to the well‐established impact of long‐term immunosuppressive therapy [70].

Therefore, our current and future efforts focus on the development of autologous models, incorporating endothelial, stromal, tumoral, and immune components from a single patient. This approach may allow for a more physiologically relevant representation of cellular interactions and could improve the translational relevance of the system in future applications. In summary, these findings underscore the importance of carefully accounting for all major cellular and structural components when designing immune‐responsive tumor models in vitro to study the interplay of angiogenesis, desmoplasia, immune activity, and the resulting tumor responses.

## 5 Experimental Section

### 5.1 Cell Culture

GFP‐HUVEC (Human Umbilical Vein Endothelial Cells) were purchased from PeLO Biotech (Planegg, Germany) (cat. no. PB‐CAP‐0001GFP (no RRID available)) on 25.05.2021 and cultured up to passage 5 in VascuLife VEGF Endothelial Medium (VascuLife VEGF Endothelial Medium Complete Kit, Lifeline Cell Technology, LLC, Oceanside, USA). Normal Human Dermal Fibroblasts (nHDF) were purchased from Promo Cell (Heidelberg, Germany) (cat. no. C‐12302 (no RRID available)) on 19.01.2022 and were cultured up to passage 6 in FibroLife S2 Fibroblast Medium (FibroLife S2 Fibroblast Medium Complete Kit, Lifeline Cell Technology, LLC, Oceanside, USA). The human TNBC cancer cell line HCC1937 (RRID: CVCL_0290) was generously provided by the Institute of Pathology (RWTH University Hospital Aachen, Germany) and was cultured in RPMI 1640 (Invitrogen, Darmstadt, Germany) with 10% FCS (Fetal Calf Serum; Invitrogen; Darmstadt, Germany) and 1% Pen/Strep (Penicillin‐Streptomycin; Invitrogen, Darmstadt, Germany). All cell lines were negative for mycoplasma with RT‐PCR and other contaminations during regular tests. Tumor spheroids were formed according to previously described protocols [20]. Briefly, HCC1937 cells were co‐seeded with nHDF in a ratio of 1:4 (4000:16 000 cells) in 96 ultra‐low attachment well plates (ULA plates, CLS7007, Corning, New York, USA). Spheroids were cultivated for 7 days before they were introduced into the bioreactors. To determine changes in spheroid sizes, HCC1937 cells were labeled with DiI (Vybrant DiI, V22885, Cell‐Labeling Solution, Invitrogen, Waltham, Massachusetts, USA) according to instructions provided by the manufacturer. We selected these cell lines based on their well‐established roles in modeling vascularized tumor microenvironments. The HCC1937 TNBC line was chosen due to its ability to promote vascularization and its clinical relevance as an aggressive breast cancer model. The 1:4 ratio was chosen based on an optimization screen, which was performed by our group. There, we tested multiple TNBC cell lines, including HCC1937, in combination with several nHDF ratios (1:1, 1:2, 1:4). For HCC1937, the 1:4 ratio yielded the most robust tumor spheroid architecture, meaning the highest circularity and compactness, strongest ECM deposition, and highest CAF‐marker expression (α‐SMA, FAP) [71]. Based on these outcomes, we adopted the 1:4 ratio for HCC1937 in all subsequent experiments. NHDF were used to model de novo CAF activation within the tumor microenvironment, offering a stable starting population that can acquire CAF‐like features when co‐cultured in 3D with TNBC cells in our system (increased α‐SMA and FAP expression, enhanced matrix deposition and organization, and higher contractility). This progression mirrors how resident fibroblasts become activated within tumors. Finally, GFP‐HUVEC were used because their intrinsic fluorescence enables real‐time visualization of endothelial network formation and dynamics by live microscopy. HUVEC are widely considered the gold standard for in vitro vascularization studies due to their robust angiogenic behavior and physiological relevance.

### 5.2 Bioreactor Casting and Dynamic Cultivation

The experimental setup followed our previously developed procedure to obtain tumor spheroids with a perfusable vasculature under dynamic cultivation. Single‐inlet bioreactors were chosen to conduct these experiments [20]. Briefly, the bioreactors were manufactured from polyether‐ether ketone (PEEK) via CNC milling at the RWTH University Hospital (Aachen, Germany). Borosilicate glass coverslips were used as optical windows into the bioreactors (dimensions: 24 × 40 mm) (BRAND GmbH + Co KG, Wertheim, Germany). These were coated with a collagen‐fibrinogen hydrogel blend, a day prior to the assembly of the bioreactors. While assembling the bioreactors, coverslips were fixed to the bioreactor bodies with silicone seals that were previously cast in 3D printed molds at the RWTH University Hospital (Aachen, Germany). These components were assembled with stainless steel screws (Conrad Electronic, Bonn, Germany). To enable fluid flow through the bioreactors, 18G Leur‐lock needles were placed into both the in‐ and outlets of the bioreactors (length: 12.7 mm; inner diameter: 0.88 mm) (Nordson EDF, Westlake, Ohio, USA). The needle sealing was ensured using NBR 90 O‐rings (inner diameter: 0.88 mm, cord thickness: 0.92 mm) (Sealware International Dichtungstechnik GmbH, Limburg, Germany). Oxygen‐permeable, medical‐grade silicone tubing (inner diameter: 1.6 mm) was connected to the bioreactors via barbed to male Leur‐lock adapters (Darwin Microfluidics, Paris, France), which were added to the ends of the needles. Silicone tubing was furthermore connected to PharMED BPT 3‐stop tubing via tubing‐to‐tubing connectors (both Darwin Microfluidics, Paris, France). Before use, all components were autoclaved.

Before filling the bioreactors with cells and hydrogel material, channel geometries were positioned on top of the glass coverslips with sterile filtered 10% (w/v) gelatin (gelatin from porcine skin) through an 18G needle (B. Braun SE, Melsungen, Germany). Additional gelatin was used to fill both inlets and outlets of the bioreactors. Tumor spheroids that were previously cultivated for 7 days were embedded within the gelatin strands in a cellularized collagen–fibrinogen–hydrogel mixture, the composition of which was described in detail in our previous study [20]. Briefly, 10^6^ GFP‐HUVEC as well as 10^6^ nHDF were resuspended in the collagen‐fibrinogen hydrogel mixture. Following the embedding of the spheroids, the hydrogel was left to solidify at RT for 30 min, after which the remaining volume of the bioreactors was filled with 2.4 mL of hydrogel blend without cells. The bioreactors were then sealed and placed into an incubator for 2 h at 37°C, allowing for the gelatin to liquefy. To remove the liquid gelatin, channels were flushed with warm DPBS. The channels were then injected with GFP HUVEC in a concentration of 2 × 10^7^ cells/mL in a volume of 200 µL for each bioreactor. Bioreactors were left in an incubator for another 2 h, allowing for the endothelial cells to adhere to the channels.

For the generation of fluid flow through the bioreactors, an Ismatec peristaltic pump with independent channel control was used (4 independent channels; 12 rollers) (Darwin Microfluidics, Paris, France). The media reservoir consisted of 100 mL glass bottles with HPLC Duran GL 45 connection system caps (DURAN Group GmbH, Wertheim, Germany) and filled with growth media tailored to the cell types contained within the bioreactors (33% RPMI 1640, 33% VascuLife, 33% FibroLife, and 1% antibiotic‐antimycotic solution). The total FCS content of the media was set to 5%. After completing the setup, bioreactors were connected to fluid flow with a maximum of four bioreactors per loop. The flow speed was set to 0.1 mL/min. Vascular network formation was observed for 7 days, with image acquisition of the channels, vasculature, and spheroids performed every two days starting from day three of cultivation. A media change was performed every two days, replacing 50% of the media.

### 5.3 Immune Cell Isolation and Injection

Peripheral blood mononuclear cells (PBMC) were isolated from three anonymized, healthy female donors’ whole blood in the form of leukotraps obtained from the Department of Transfusion Medicine (RWTH University Hospital Aachen, Germany). All donors provided informed consent allowing the use of donated blood for research purposes. No identifiable information was available. Individual experiments were performed with PBMC from a single donor at a time. Briefly, a standard density gradient centrifugation with Ficoll (Cytiva Ficoll‐Plaque PREMIUM, 11753219, Thermo Fischer Scientific, Langerwehe, Germany) was performed to separate the PBMC from other blood components [72]. The isolated cells were immediately frozen in 90% donor serum and 10% DMSO at a concentration of 1 × 10^8^ cells/mL. On the day of PBMC administration, thawed cells were prepared by suspending 4 × 10^7^ PBMC in 50 mL serum‐free RPMI 1640 medium, supplemented with 25 µL of DiD cell‐labeling solution. After labeling, the cells were washed twice with calcium‐ and magnesium‐free DPBS (1X) (Gibco, Grand Island, New York, USA), followed by a final cell count. The PBMC were then resuspended in serum‐free RMPI 1640 at a concentration of 8 × 10^6^ cells/mL, ready for subsequent use. On day 8 of dynamic cultivation, fluid flow was temporarily halted, and the inlet tubing of the first bioreactor in each injection loop was detached. A 1.5 mL PBMC solution was injected into each loop with a 1 mL Leur‐lock syringe (B. Braun Omnifix F Solo, B. Braun, Melsungen, Germany) in two rounds of 750 µL, amounting to 3 × 10^6^ PBMC per bioreactor (4 bioreactors per loop). This concentration was intentionally chosen to exceed physiological PBMC levels in peripheral blood (typically 2 × 10^6^ cells/mL in healthy female donors), as cell loss was expected within the tubing and connectors [73]. Once flow was restarted following injection, we anticipated that this approach would compensate for the initial loss and result in a physiological number of PBMC within the bioreactors. After 5 h, the injected bioreactors were reconnected to the fluid flow. Bioreactors were imaged at the following time points post‐PBMC injection: 4 h, 24 h, 4 days, 7 days. Tumor spheroid growth was tracked by analyzing the fluorescent signal emitted by Dil‐labeled cancer cells, using AxioVision 4.8 software to measure the spheroid cross‐sectional area. After 15 days of dynamic cultivation, hydrogels were retrieved from the bioreactors for further histological analysis.

### 5.4 Histological Analysis

For histological analysis, hydrogels were fixed in 4% PFA for 24 h, washed with PBS, and embedded in TissueTek O.C.T. Compound (Sakura Finetek, Staufen im Breisgau, Germany). The samples were snap‐frozen, stored at ‐80°C, and sectioned into 10 µm thick slices, which were mounted onto microscopy slides. Immunofluorescent staining was performed using primary and secondary antibodies according to established protocols [20]. The following anti‐human primary antibodies that were used can be found in Table S1. 5% normal donkey serum (ab7475, Abcam, Cambridge, UK) or 12% bovine serum albumin (BSA) was used for blocking purposes. Primary antibodies were then combined with the corresponding secondary antibodies that can be found in Table S2. DAPI (ThermoFischer, Waltham, Massachusetts, United States) was used for nuclear staining.

For the analysis of the prepared sections, five independent bioreactors were examined per condition. From each bioreactor, six slides were generated, each containing up to two 8‐µm histological sections taken from different depths to minimize sampling bias. While these thin sections represent limited optical sampling planes, they are intended to assess cellular composition and marker expression rather than to capture the full 3D extent of the vascular network. 3D vascular architecture was assessed using complementary imaging approaches with increased imaging depth, such as two‐photon laser‐scanning microscopy. From each section, six regions of interest (ROIs) were acquired, and all ROIs from a given bioreactor were averaged to yield one biological replicate (n = 5 per condition). For the analysis of stained spheroids, all quantifications were normalized to the number of nuclei per spheroid and are expressed as percentages to mitigate potential confounding effects of spheroid compaction. To analyze tumor cell invasion, CK8^+^ cells were classified as invasive if they were detected 15–215 µm away from the spheroid boundary that was set by drawing a contour around the spheroid core. This distance was selected to exclude cells still associated with the spheroid surface and to capture only those that had migrated into the surrounding matrix.

### 5.5 Two‐Photon Imaging

Samples were mounted on a 50 mm glass‐bottom petri dish (Mattek, MA, USA, #P50G‐1.5‐14‐F), filled with 250 µL PBS, and covered with a #1.5 coverslip. Imaging was performed on a STELLARIS DIVE FALCON multiphoton microscope (Leica Microsystems GmbH, Wetzlar, Germany) equipped with a motCORR HC IRAPO L 25×/1.00 W water‐immersion objective. Excitation was set to 840 nm, and detection was carried out simultaneously for SHG (415–425 nm), DAPI (440–490 nm), GFP (520–560 nm), and DiD (650–700 nm). All samples were acquired with identical settings, imaging the entire section as a mosaic. Pixel size was 432 × 432 nm with a 1024 × 1024‐pixel format per tile, and z‐stacks were acquired with a 1.5 µm step size. Mosaic dimensions ranged from 4–16 mm^2^ depending on sample size. For quantification, a 1.3 mm^2^ region excluding the tumor area and sample margins was analyzed. Collagen quantification was performed on maximum intensity projection (MIP) images of the SHG channel. Images were thresholded (=3), and particle area, mean intensity, and integrated intensity were measured using the Analyze Particles function in Fiji with a 2‐pixel cutoff [74]. Average particle size was calculated by dividing the total collagen area by the number of detected objects. Area fraction was obtained by dividing the collagen‐positive area by the analyzed region and multiplying by 100, and integrated density was computed as the product of mean particle intensity and total collagen area.

### 5.6 Flow Cytometry Analysis

Hydrogel samples from bioreactors were extracted at 24 h, 4 days, and 7 days after PBMC perfusion. Samples were washed with DPBS three times, after which they were digested with nattokinase (100 FU/mL, HY‐P2373, Hycultec, Beutelsbach, Germany) [75]. Tissues were digested for 15–20 min at 37°C with gentle pipetting performed every 5 min. After tissue homogenization, the cell suspension was passed through a 40 µm cell strainer (542040, Greiner Bio‐One, Kremsmünster, Austria), washed twice with FACS buffer (DPBS (1×), 5% FCS, and 1 mM EDTA), and stained for flow cytometry analysis (Table S3). Flow cytometry analysis was performed with a LSR Fortessa Cell Analyzer (BD Bioscience, Franklin Lakes, New Jersey, USA). Data analysis was performed with FlowJo v10.8 Software (BD Bioscience, Franklin Lakes, New Jersey, USA).

### 5.7 Inflammatory Cytokine Profiling

The expression levels of inflammatory cytokines were analyzed using a human inflammation antibody array membrane (ab134003, Abcam, Cambridge, UK). For each condition, culture media from 12 individual bioreactors were pooled in equal volumes after 15 days of cultivation. Membrane preparation and incubation were performed according to the manufacturer's instructions. Chemiluminescent signal detection was carried out using a ChemiDoc imaging system (Bio‐Rad, Hercules, CA, USA), with CCD images acquired at an average exposure time of 2.4 s.

### 5.8 Cytokine Stimulation Assays

Wound healing assays were performed for the analysis of cytokine‐induced cancer cell migration in 2D. HCC1937 cells were cultured as described in Section 5.1 and seeded in 96‐well plates at a density of 3.5 × 10^6^ cells/mL, with 3.5 × 10^5^ cells per well. After 24 h and the formation of a confluent monolayer, cells were pretreated with Mitomycin C (5 µg/mL in RPMI‐1640 (M5353, Sigma‐Aldrich, St. Louis, Missouri, USA)) for 2 h to inhibit proliferation and thereby ensure that subsequent wound closure reflected cancer cell migration, rather than cell division. In parallel, non‐treated controls were included to assess the contribution of proliferation to the overall wound closure. Linear wounds were generated with 100 µL pipette tips, after which the cell monolayers were washed twice with DPBS to remove debris. Subsequently, the media was supplemented with individual cytokines or cytokine combinations and added to the wells.

The following recombinant human cytokines were tested: IL‐8 (Hz‐1318), IL‐6 (Hz‐1019), MCP‐1/CCL2 (Hz‐1334) (cytokines purchased from Proteintech, Rosemont, Illinois, USA), MIP‐1α (300‐08), and TIMP‐2 (410‐02) (cytokines purchased from Thermo Fischer Scientific, Langerwehe, Germany). Each cytokine was added at concentrations of 5, 15, and 50 ng/mL to cover sub‐threshold to nearly saturated concentrations [76]. To assess combinatorial effects, cytokines were also applied in pairwise, triple, and quadruple combinations at 15 ng/mL per cytokine. Corresponding vehicle controls (PBS + 0.1% BSA (Bovine Serum Albumin)) and untreated controls were accounted for in the experiments. Images of wound closures were acquired using an inverted Zeiss Axio Observer fluorescent microscope immediately after implementing the wound (t = 0 h) and at 4, 8, 12, 16, 24, and 30 h. The wound area was quantified using the “MRI Wound Healing Tool” plugin in Fiji. Here, the percentage of wound closure over time was calculated relative to the initial wound area.

For the assessment of cancer cell invasion in 3D, HCC1937 and nHDF heterospheroids were cultured as described in section 5.1. After 7 days of culture, spheroids were embedded within the collagen‐fibrinogen hydrogel with GFP HUVEC and nHDF, replicating the cell compositions and ratios that were applied in the vascularized bioreactor model within 96‐well plates. The embedded spheroids were cultured for 4 days in medium supplemented with the cytokines and cytokine combinations identified as most effective in promoting cancer cell migration in the foregoing 2D wound healing assays. A full medium change was performed after 48 h. Hydrogels were later removed from the wells, fixed in 4% PFA, and cryosectioned. Immunofluorescent staining for CK8 was performed on the sections to visualize tumor cell invasion from the spheroid periphery.

### 5.9 Fluorescence Microscopy

Image acquisition was performed using an Axio Imager.M2 microscope with an AxioCamMRm Rev.3 camera (Zeiss, Oberkochen, Germany). For observing spheroid growth, vascular network expansion, and immune cell migration, an EC Plan‐Neofluar objective was used with a magnification of 5x and a numerical aperture of 0.16. Average exposure times of the GFP signal for both GFP‐HUVEC within the channels and the vascular networks were set at 100–200 ms. DIL (tumor spheroids) and DID signal (PBMC) were acquired with fixed exposure times of 1000, and 1500 ms, respectively. The analysis of vascular network formation and remodeling was performed with AngioTool. Specifically, the elimination of fore‐ and background small particles was set to values of 256 for the removal of small particles and 200 for the filling of holes. Fluorescent staining images were acquired using the same microscope with an EC Plan‐Neofluar objective with a magnification of 10x and a numerical aperture of 0.3. Image processing was conducted using FIJI or AxioVision 4.8 software.

### 5.10 Statistical Analysis

All data are presented as mean values with standard deviations. Graphical representations were created using GraphPad Prism 10 (GraphPad Software, Inc., San Diego, CA, USA). Levels of significance are indicated in each figure. Statistical analysis was performed using the Kruskal‐Wallis one‐way ANOVA test, assuming non‐Gaussian data distribution. The number of bioreactors used for each type of analysis is represented by individual data points.

## Author Contributions

F.D. and F.K. conceptualized the study. F.D., F.K., and T.L. supervised the study. A.R., F.D., and F.K. designed the experiments. A.R., R.D., and O.W. conducted 2D and 3D cell culture, as well as bioreactor assembly and casting. A.R. performed dynamic cultivation of vascularized tumors, immune cell isolation and injection, and histological analysis. H.F. contributed to establishing the flow dynamics and modelling the shear stress. M.A.S.T. performed flow cytometry experiments and analyzed the data. A.R. and D.K. performed the acquisition and analysis of two‐photon images. A.R., F.D., and F.K. analyzed the data. A.R., T.L., and F.D. designed the figures. A.R. performed statistical analysis and drafted the original manuscript. All authors read, revised, and approved the final version of the manuscript.

## Funding

DFG: GRK2375 (Project number: 331065168)

## Conflicts of Interest

The authors declare no conflicts of interest.

## Supporting information




**Supporting File**: advs74267‐sup‐0001‐SuppMat.docx.

## Data Availability

The data that support the findings of this study are available on request from the corresponding author. The data are not publicly available due to privacy or ethical restrictions.
