# Supplementary material for: Allogeneic Immune Cell Perfusion Inhibits the Growth of Vascularized 3D In Vitro Tumor Models, Induces Vascular Regression and Desmoplasia, but Promotes Tumor Cell Invasion
Source: Adv Sci (Weinh). 2026 Feb 6;13(22):e14361. doi: 10.1002/advs.202514361 (PMC13088334; doi:10.1002/advs.202514361)
Supplement: Supplementary file 1 — Supporting File: advs74267‐sup‐0001‐SuppMat.docx. [file ADVS-13-e14361-s001.docx]

Supporting Information

Allogeneic Immune Cell Perfusion Inhibits the Growth of Vascularized 3D In Vitro Tumor Models, Induces Vascular Regression and Desmoplasia, but Promotes Tumor Cell Invasion

*Alexandra Raab^1^, Rasika Daware^2^, Marcelo A.S. de Toledo^3^, Oskar Weber^1^, Dimitris Kapsokalyvas^4^, Twan Lammers^2^, Horst Fischer^5^, Federica De Lorenzi^1,6*^, Fabian Kiessling^1*^*

1. Institute for Experimental Molecular Imaging (ExMI), RWTH Aachen University Hospital, Aachen, Germany

2. Department of Nanomedicine and Theranostics, Institute for Experimental Molecular Imaging (ExMI), RWTH Aachen University Hospital, Aachen, Germany

3. Department of Hematology, Oncology, Hemostaseology and Stem Cell Transplantation, Faculty of Medicine, RWTH Aachen University, Aachen, Germany

4. Interdisciplinary Center for Clinical Research (IZKF), RWTH Aachen University Hospital, Aachen, Germany

5. Department of Dental Materials and Biomaterials Research (ZWBF), RWTH Aachen University Hospital, Aachen, Germany

6. Department of Medical BioSciences, Radboud University Medical Center, Nijmegen, the Netherlands

E-mail: fdelorenzi@ukaachen.de; fkiessling@ukaachen.de

These supplementary figures provide additional histological and quantitative data illustrating the activation of apoptotic pathways in tumor, endothelial, and stromal compartments, and the baseline distribution of immune cell subsets within donor PBMC. They expand on the main findings by showing how immune cells interact with the vasculature and stroma, resulting in pronounced remodeling of the tumor microenvironment.


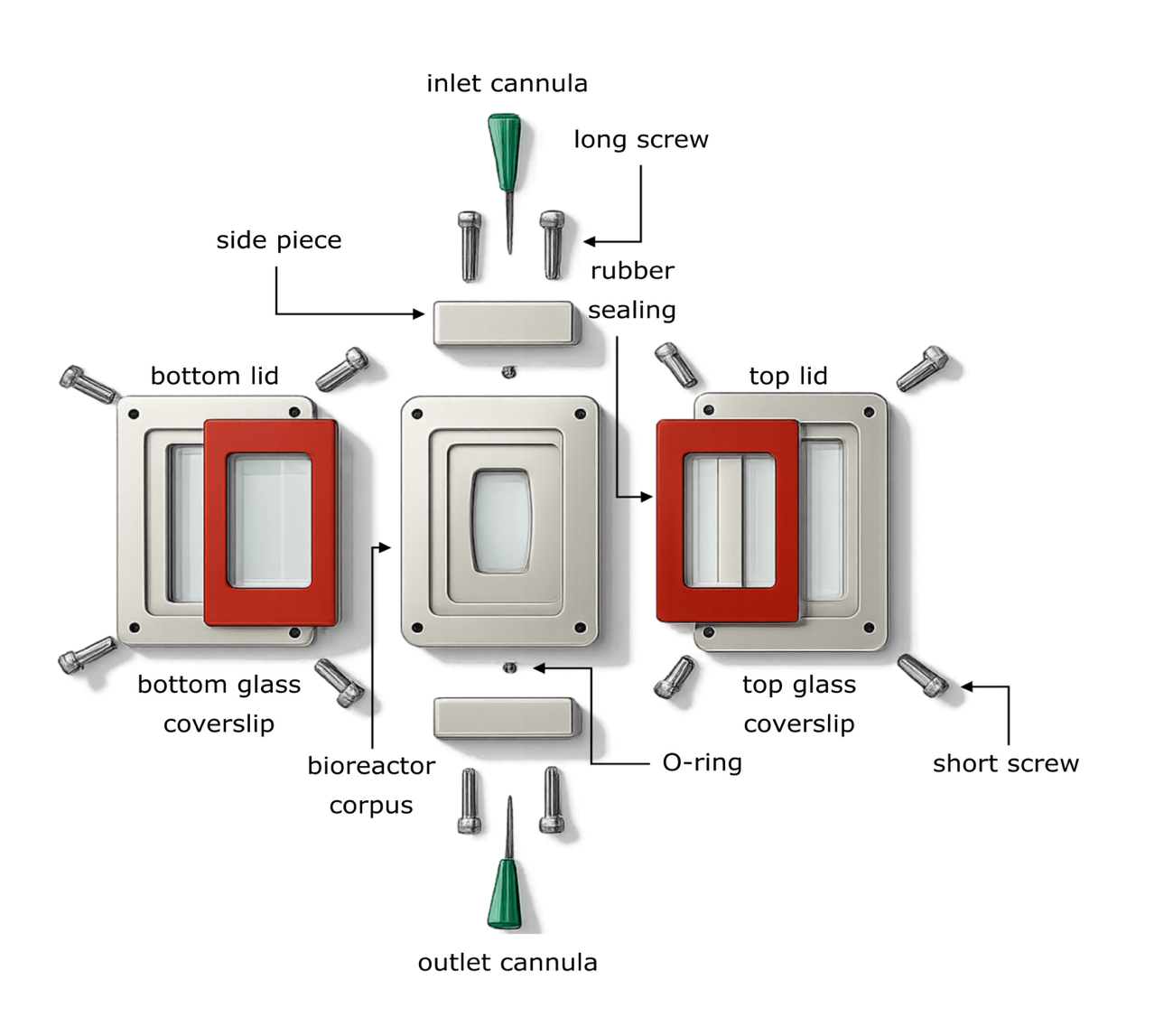


**Figure S1. Expanded view of the mesoscopic bioreactor model.** Exploded representation of the individual in-house fabricated PEEK bioreactor components, featuring optical windows for imaging applications. (Rendered with Illustrae.co)

*
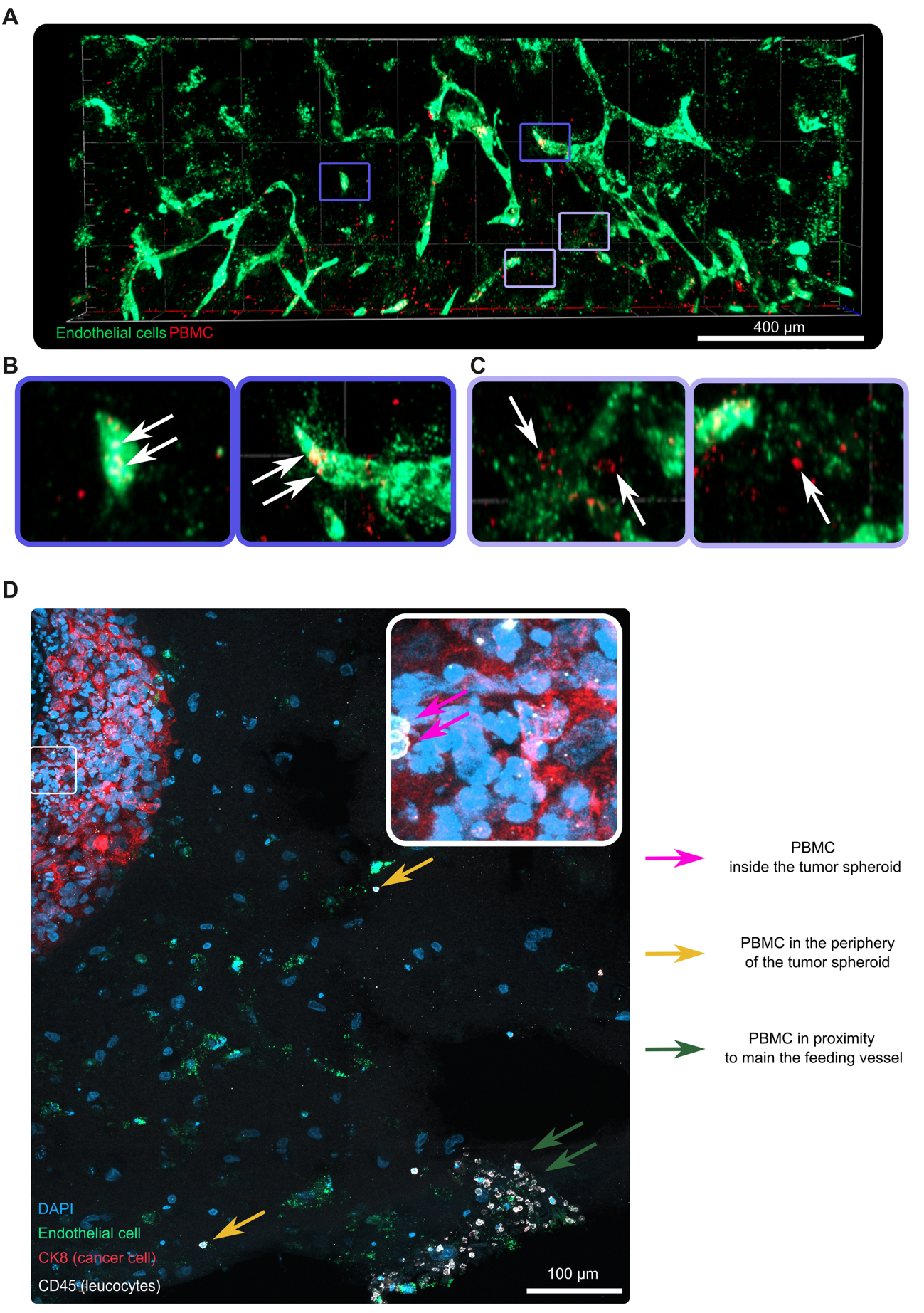
*

**Figure S2. Spatial localization of PBMC relative to endothelial cells and tumor spheroids. (A)** Confocal microscopy reveals PBMC localized within vessels containing dying endothelial cells (highlighted by circles with white borders). Additionally, PBMC are also found next to dead endothelial cells (highlighted by rectangles with white border), showing that they remain at the site of the endothelial cell following its death. **(B)** Magnified views of the regions marked by circles in (A), showing the presence of PBMC in vessels with dying endothelial cells with white arrows pointing towards the PBMC. **(C)** Magnified views of the regions marked by rectangles in (A), showing PBMC co-localization with dead endothelial cells with white arrows pointing towards the PBMC. **(D)** Confocal tile-scanned image (25x) of a 40 µm-thick cryosection illustrating distinct PBMC positions relative to the tumor spheroid. CD45^+^ PBMC are found near the feeding vessel (green arrows), accumulating along the spheroid periphery (yellow arrows), and in some cases infiltrating the tumor spheroid, as shown by two internalized CD45^+^ cells (pink arrows). A magnified image in the top right corner highlights the intratumoral immune cells.


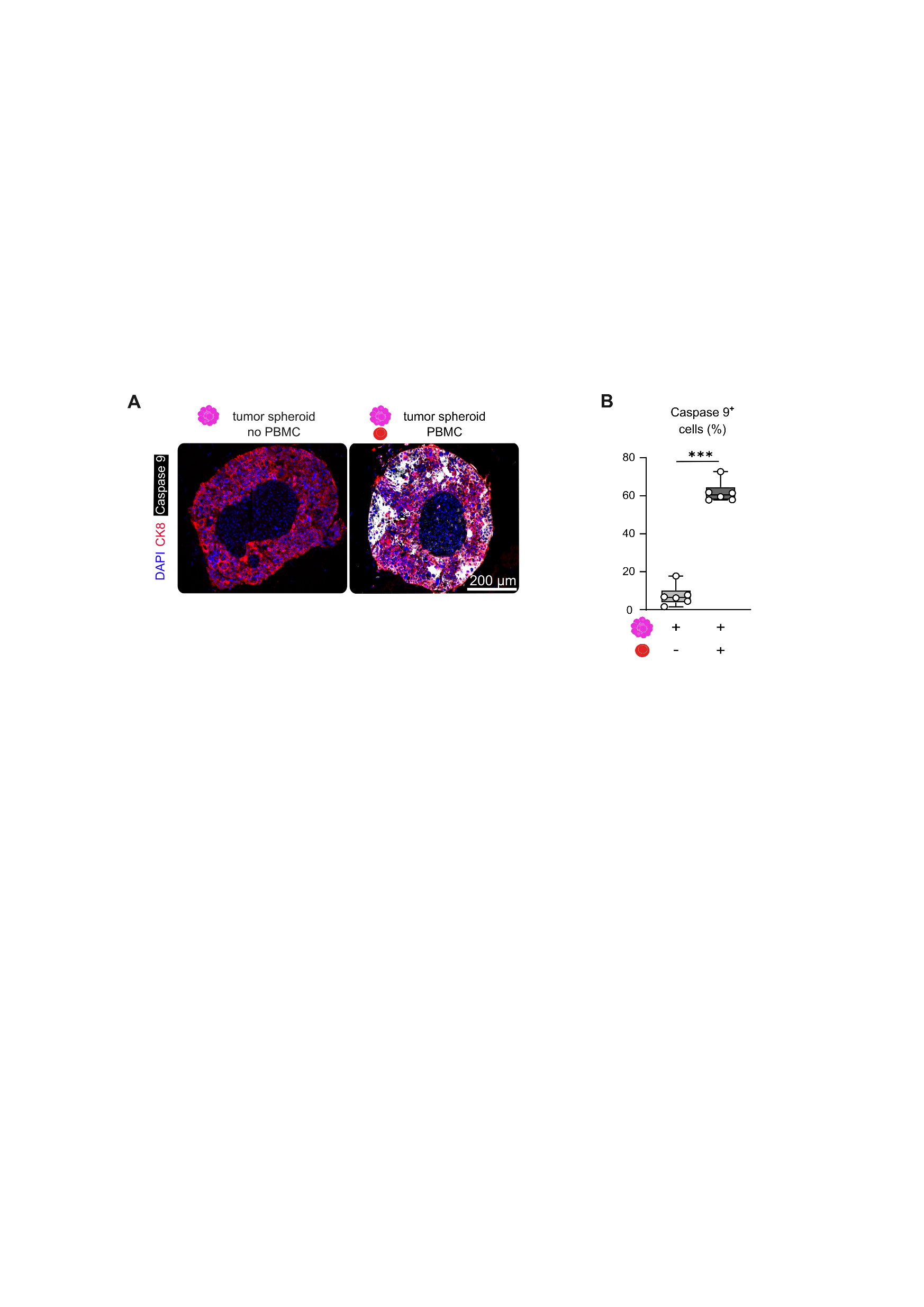


Figure S3. Further histological characterization of PBMC-tumor interactions. (A) Representative fluorescence microscopy images of tumor spheroids after 15 days of dynamic cultivation. They show increased levels of caspase 9 in spheroids cultured in bioreactors that were perfused with PBMC compared to those in PBMC-free bioreactors. Spheroids were co-stained with CK8 to distinguish CK^+^ cancer cells and CK^-^ fibroblasts within the spheroids. (B) Quantification of immunofluorescence staining reveals a significant elevation of caspase 9 levels within tumor spheroids, indicating the initiation of apoptotic pathways following PBMC perfusion of bioreactors. Data are displayed as mean ± SD of n = 6 spheroids per group. Statistical significance was assessed using a two-tailed Mann-Whitney test, assuming a non-Gaussian distribution of the data*.* P-values: *** <0.01.


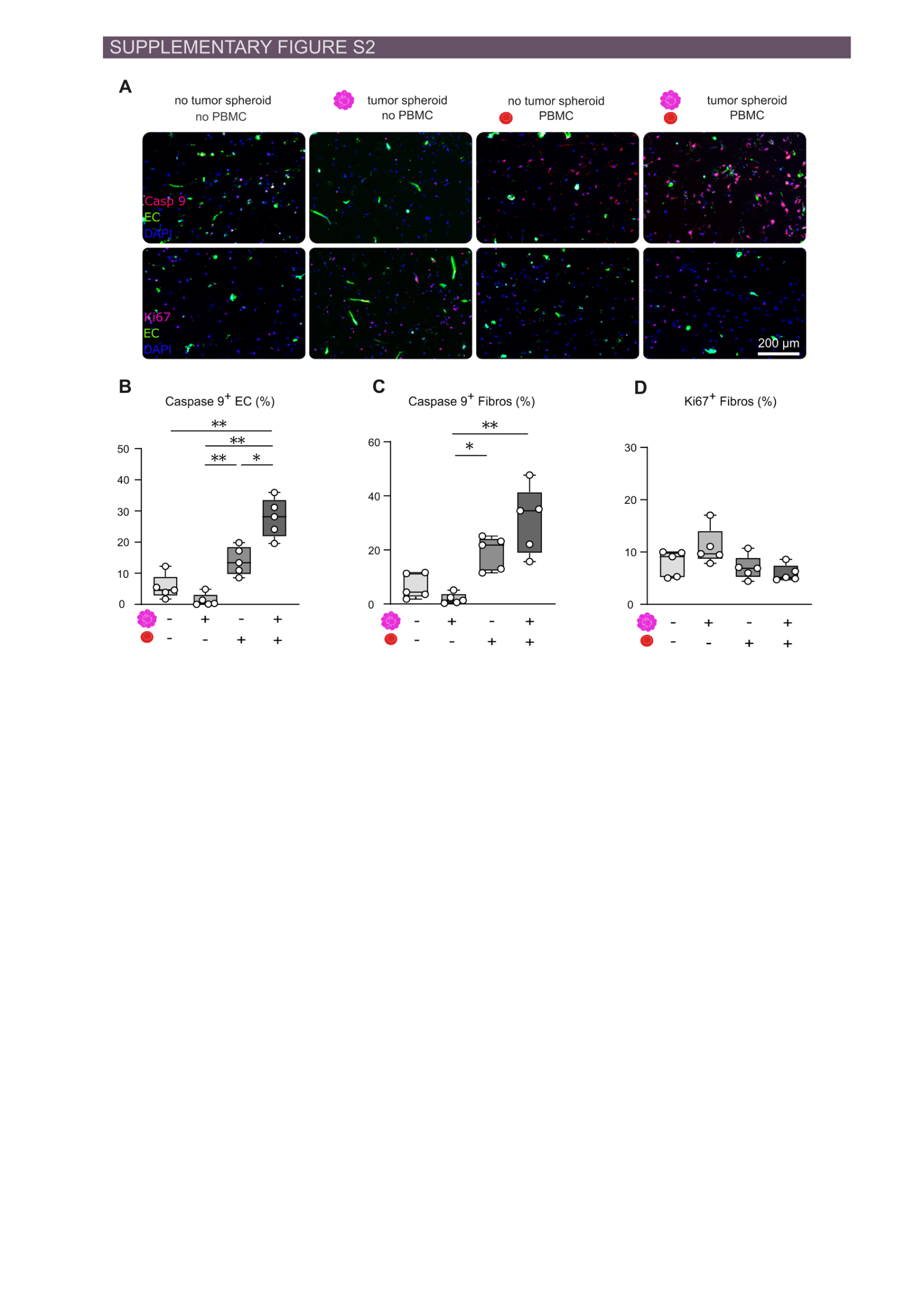


Figure S4. Further histological characterization of PBMC-stroma interactions within the tumor microenvironment. (A) Representative fluorescence microscopy images illustrate the space surrounding tumor spheroids after 15 days of dynamic cultivation. They show increased caspase 9 expression in both endothelial cells and fibroblasts, along with reduced Ki67 expression in fibroblasts following PBMC perfusion. (B-D) Quantification of immunofluorescence staining reveals elevated levels of caspase 9, signifying the initiation of the apoptotic pathway in endothelial cells (B) and fibroblasts (C), following PBMC perfusion. In contrast, the percentage of proliferative fibroblasts decreases (D). Data are displayed as mean ± SD n = 5 bioreactors per group. Statistical significance was assessed using a Kruskal-Wallis ANOVA test followed by Dunn´s multiple comparison correction, assuming a non-Gaussian distribution of the data*.* P-values: * <0.05, ** <0.01.

**
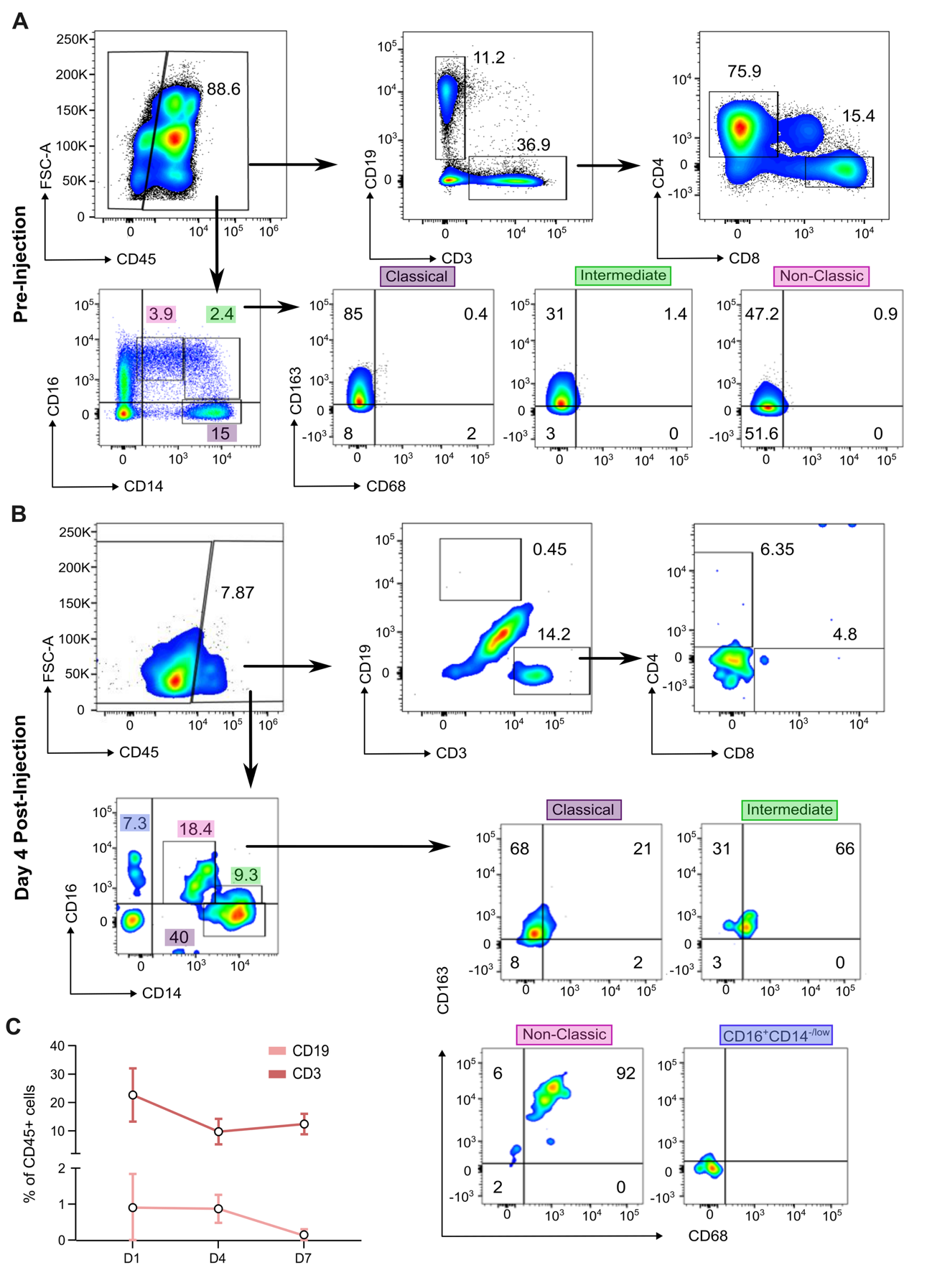
**

**Figure S5. Flow cytometry reveals a shift from lymphoid toward myeloid immune cell compositions following allogeneic PBMC engraftment in the bioreactors. (A)** Exemplary gating strategy used to characterize PBMC subpopulations before injection. PBMC were identified as CD45⁺ cells, with subsequent discrimination of lymphoid subsets, including B cells (CD19⁺) and T cells (CD3⁺; CD4⁺ helper T cells; CD8⁺ cytotoxic T cells). Myeloid cells and their monocytic subsets were identified by CD14 and CD16 expression, followed by subgating with CD68 and CD163. **(B)** Gating strategy performed on cells recovered from the bioreactors 4 days after PBMC administration. **(C)** Quantification of CD3⁺ T cells and CD19⁺ B cells recovered at 1, 4, and 7 days after PBMC injection, expressed as a percentage of CD45⁺ cells. Both lymphoid populations progressively decline over time, with CD19⁺ B cells nearly absent by the endpoint. Data are shown as mean ± SD (n = 3 bioreactors).


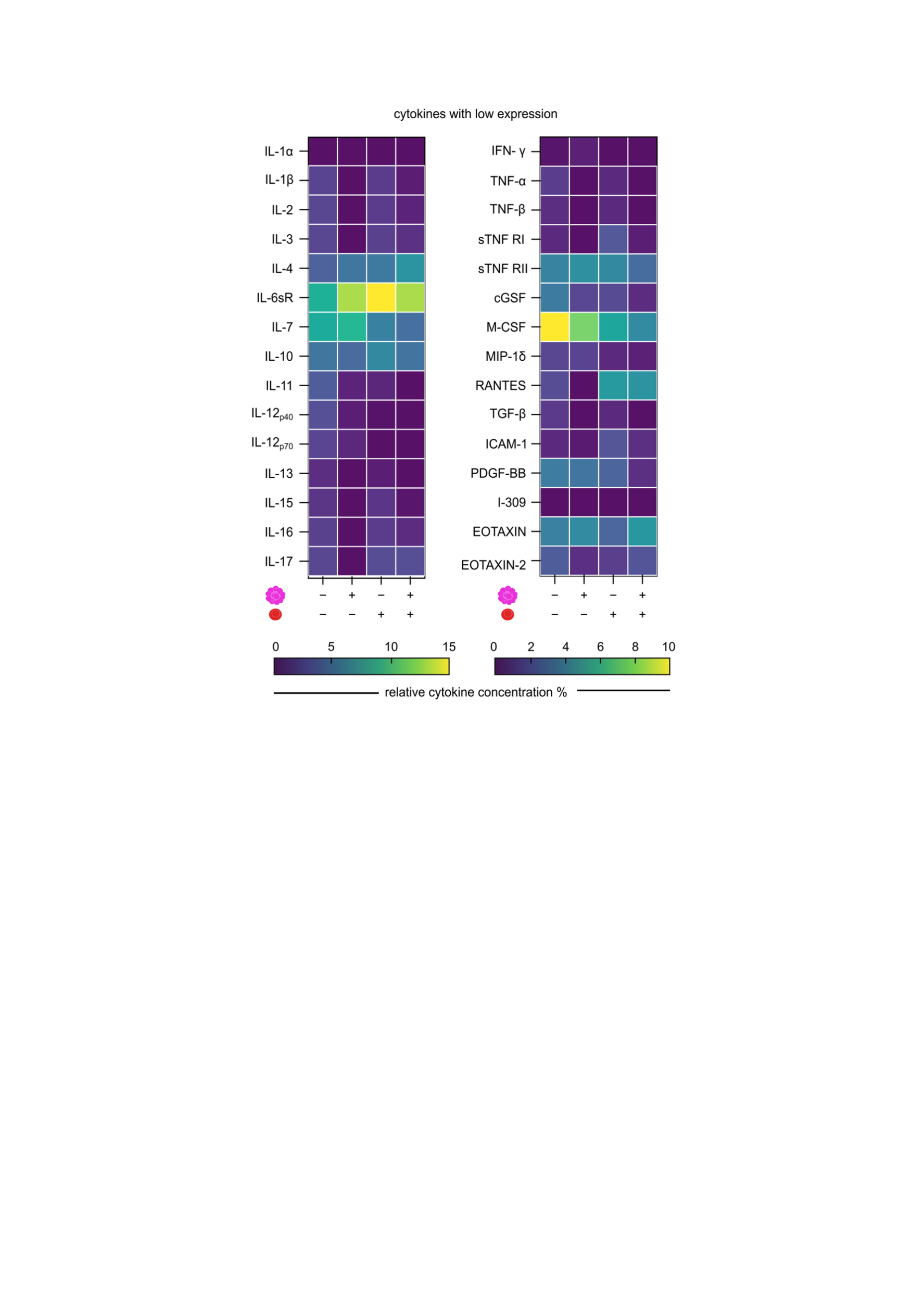


**Figure S6. Relative concentrations of inflammatory cytokines with low expression.** Cytokine profiles were extracted from media that was pooled from n = 3 different experiments with n = 4 bioreactors per experiment and condition after 15 days of cultivation. These cytokines remained at minimal or even undetectable levels across all groups, indicating an absence of cytokines typically associated with the activation of cytotoxic T cell pathways.


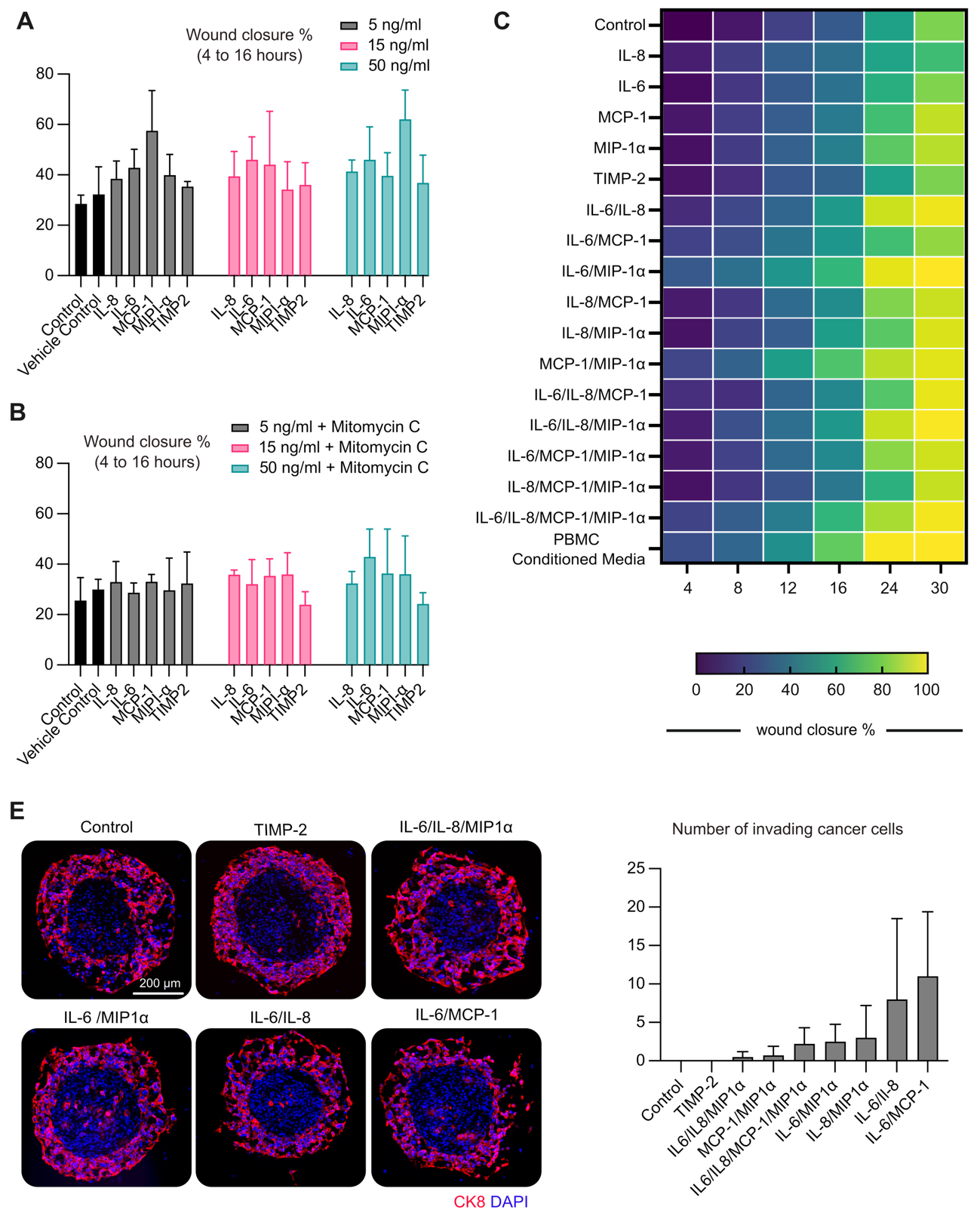


**Figure S7. Cytokine-mediated modulation of tumor cell migration and invasion across 2D and 3D cultures. (A)** Wound closure kinetics of HCC1937 TNBC cells exposed to individual cytokines at 5, 15, and 50 ng/ml. Based on the Δ-closure from 4 to 16 hours, the added cytokines increased wound closure relative to controls. (n = 4) **(B)** Migration-specific wound closure dynamics after mitomycin C pretreatment. Between 4 and 16 hours, MCP-1 and MIP-1α were most effective at promoting tumor cell migration across low and high cytokine concentrations, while IL-6 induced strong migration at primarily at 50 ng/ml. In contrast, TIMP-2 consistently inhibited migration across all tested conditions. (n = 4) **(C)** Heatmap showing 30-hour wound closure dynamics of mitomycin C-pretreated HCC1937 cells exposed to cytokines and cytokine combinations at 15 ng/ml. Across combinations, IL-6 emerged as a key synergistic amplifier, particularly with IL-8 and MIP-1α. PBMC-conditioned media also produced high closure rates over the 30-hour period. (n = 4) **(D)** Representative fluorescence images of tumor spheroids after 4 days in endothelial cell- and fibroblast-laden hydrogels under static conditions. Each pair of images (upper and lower rows) illustrates conditions resulting in absent, sparse, or increased tumor cell dissemination. (n = 5-6 spheroids) **(E)** Quantification of CK8⁺ tumor cell dissemination between 15 and 215 µm from the spheroid boundary. IL-6/IL-8 and IL-6/MCP-1 combinations induced the highest levels of tumor invasion within the cellularized hydrogel matrix. Data in (A) and (B) are displayed as mean ± SD of n = 4 wells per group. Data in (D) is displayed as mean ± SD of n = 5-6 spheroids per group.

**Table S1.** List of primary antibodies used for immunofluorescent staining of histology samples

| Antibody | Company | Catalogue Number |
| --- | --- | --- |
| Anti-Cytokeratin 8 | Abcam, Cambridge , UK | ab9023 |
| Anti-Cytokeratin 8 | Abcam, Cambridge , UK | ab53280 |
| Anti-Ki67 | Abcam, Cambridge , UK | ab15580 |
| Anti-Caspase-9 | Abcam, Cambridge , UK | ab32539 |
| Anti-CD45 | Abcam, Cambridge , UK | ab8216 |
| Anti-CD8 | Abcam, Cambridge , UK | ab237709 |
| Anti-CD68 | Abcam, Cambridge , UK | ab213363 |
| Anti-VEGFR2 | Dianova, Hamburg, Germany | CYT-28770 |
| Collagen I | Thermo Fischer, Waltham, Massachusetts, United States | PA5-95137 |
| Anti-Integrin α_v_ß_3_ | Bioss, Woburn, Massachusetts, United States | bs1310R |
| FAP | Santa Cruz, Dallas, Texas, United States | sc-65398 |
| Human Caspase-1 | R&D Systems, Minneapolis, Minnesota, USA | MAB6215 |

**Table S2.** List of secondary antibodies used for immunofluorescent staining of histology samples

| Antibody | Company | Catalogue Number |
| --- | --- | --- |
| Donkey F(ab’)2 anti-Rat IgG (H+L)-Cy3 | Dianova, Hamburg, Germany | 712-166-153 |
| Donkey F(ab’)2 anti-Rabbit IgG (H+L)-Cy3 | Dianova, Hamburg, Germany | 711-166-152 |
| Goat IgG anti-Mouse IgG (H+L)-Cy3 | Dianova, Hamburg, Germany | 115-165-166 |
| Esel IgG anti-Kaninchen IgG (H+L)-Cy5 | Dianova, Hamburg, Germany | 711-175-152 |
| Goat IgG anti-Mouse IgG (H+L)-Cy5 | Dianova, Hamburg, Germany | 115-175-166 |
| Donkey F(ab’)2 anti-Rabbit IgG (H+L)-Alexa Fluor 488 | Dianova, Hamburg, Germany | 711-546-152 |
| Goat IgG anti-Mouse IgG (H+L)-Alexa Fluor 488 | Dianova, Hamburg, Germany | 115-225-166 |

**Table S3.** List of antibodies used for flow cytometry

| Antibody | Company | Catalogue Number |
| --- | --- | --- |
| FITC anti-human CD66b | Biolegend, San Diego, California, USA | 305103 |
| PE anti-human CD19 | Biolegend, San Diego, California, USA | 302207 |
| APC/Fire™ 750 anti-human CD68 | Biolegend, San Diego, California, USA | 333823 |
| PE/Cyanine7 anti-human CD163 | Biolegend, San Diego, California, USA | 326513 |
| anti-human CD90 APC-conjugated | Immunotools, Friesoythe, Germany | 21270906 |
| anti-human CD14 PE-conjugated | Immunotools, Friesoythe, Germany | 21620144 |
| anti-human CD16 APC-conjugated | Immunotools, Friesoythe, Germany | 21278166 |
| anti-human CD31 PE-conjugated | Immunotools, Friesoythe, Germany | 21270314 |
| CD144 (VE-Cadherin) Antibody, anti-human, REAfinity™ | Miltenyi Biotec, Bergisch Gladbach, Germany | 130-135-356 |
| CD45 Antibody, anti-human, REAfinity™ | Miltenyi Biotec, Bergisch Gladbach, Germany | 130-110-637 |
| APC-H7 Mouse Anti-Human CD8 | BD Biosciences, Franklin Lakes, New Jersey, USA | 561423 |
| BV650 Mouse Anti-Human CD3 | BD Biosciences, Franklin Lakes, New Jersey, USA | 563852 |
| APC Mouse Anti-Human CD4 | BD Biosciences, Franklin Lakes, New Jersey, USA | 555349 |
